# Genome-wide identification of B-box zinc finger (*BBX*) gene family in *Medicago sativa* and their roles in abiotic stress responses

**DOI:** 10.1186/s12864-024-10036-4

**Published:** 2024-01-24

**Authors:** Shuxia Li, Shuaiqi Guo, Xueqin Gao, Xiaotong Wang, Yaling Liu, Jing Wang, Xiaohong Li, Jinqing Zhang, Bingzhe Fu

**Affiliations:** 1 College of Forestry and Prataculture, Ningxia University, Yinchuan, China; 2 Ningxia Grassland and Animal Husbandry Engineering Technology Research Center, Yinchuan, China; 3 Key Laboratory for Model Innovation in Forage Production Efficiency, Ministry of Agriculture and Rural Affairs, Yinchuan, China; 4 Fujian Xinnong Dazheng Bio-Engineering Co., Ltd, Fuzhou, China; 5 Inner Mongolia Pratacultural Technology Innovation Center Co., Ltd, Hohhot, China

**Keywords:** Alfalfa, *BBX* gene family, Gene expression, Abiotic stress, Phytohormone, Functional verification

## Abstract

**Background:**

B-box (BBX) family is a class of zinc finger transcription factors (TFs) that play essential roles in regulating plant growth, development, as well as abiotic stress. However, no systematic analysis of *BBX* genes has yet been conducted in alfalfa (*Medica go sativa* L.), and their functions have not been elucidated up to now.

**Results:**

In this study, 28 *MsBBX* genes were identified from the alfalfa genome, which were clustered into 4 subfamilies according to an evolutionary tree of BBX proteins. Exon-intron structure and conserved motif analysis reflected the evolutionary conservation of *MsBBXs* in alfalfa. Collinearity analysis showed that segmental duplication promoted the expansion of the *MsBBX* family. Analysis of *cis*-regulatory elements suggested that the *MsBBX* genes possessed many growth/development-, light-, phytohormone-, and abiotic stress-related elements. *MsBBX* genes were differentially expressed in leaves, flowers, pre-elongated stems, elongated stems, roots and nodules, and most *MsBBX*s were remarkably induced by drought, salt and various plant growth regulators (ABA, JA, and SA). Further functional verification demonstrated that overexpressing of the *MsBBX11* gene clearly promoted salt tolerance in transgenic *Arabidopsis* by regulating growth and physiological processes of seedlings.

**Conclusions:**

This research provides insights into further functional research and regulatory mechanisms of *MsBBX* family genes under abiotic stress of alfalfa.

**Supplementary Information:**

The online version contains supplementary material available at 10.1186/s12864-024-10036-4.

## Background

Abiotic stress seriously affects the whole life process of plants, which can cause retardation of growth and development, and reduction of yield and quality [1]. With long-term evolution process, plants have developed some adaptive mechanisms to environmental stress, including regulating the coordinated expression of various stress response genes, especially transcription factors (TFs) [1]. TFs regulate the transcription of target genes through specific interactions of their DNA-binding domains with target gene promoters [2]. The zinc finger TF family is further grouped into different subfamilies according to their structural and functional diversity. The B-box (BBX) proteins are a class of zinc finger TFs with diverse function, and have received widespread attention in plants [3].

BBX TFs have specialized tertiary structures that are stabilized through binding Zn ions. BBX proteins have a general structure that consists of one or two BBX domains (near the N-terminus), and some have a CCT (CONSTANS, CO-like and TOC1) domain (near the C-terminus) [4]. The N-terminal BBX domain participates in specific protein-protein interactions, and the CCT domain plays an essential role in regulating gene transcription [5, 6]. The *BBX* gene family was first identified in *Arabidopsis thaliana*, and 32 members were divided into five groups according to the number of BBX and CCT domains [7]. Subsequently, *BBX* genes were also characterized in various plants. For example, 30 *OsBBXs*, 31 *SlBBXs*, 64 *MdBBXs*, and 25 *VvBBXs* have been identified in rice [8], tomato [9], apple [10], and grapevine [11], respectively.

BBX proteins have been shown to play a role in seedling photomorphogenesis [4], flowering [12, 13], leaf senescence [14] and the shade avoidance response [15]. Several *AtBBX* genes, including *CO*, *COL3*, *COL5* and *COL9*, can mediate flowering via the photoperiod pathway in *Arabidopsis* [16–19]. Studies have shown that BBX protein members integrate light signals perceived by plant photochromes and cryptic photoreceptors via the HY5-COP1 regulatory module, thereby influencing photomorphogenesis in seedlings [20, 21]. It has been proved that BBX proteins participate in the regulation of secondary metabolite biosynthesis, especially anthocyanins [22] and carotenoids [23]. In apple, *MdMYB1* and *MdMYB9* can positively regulate anthocyanin biosynthesis; however, MdBBX37 inhibits the binding of these genes to target genes by interacting with the MdMYB1 and MdMYB9 proteins, thus suppressing anthocyanin accumulation [24, 25]. In tomato, SlBBX20 promotes carotenoid accumulation through direct activation of the carotenoid biosynthesis enzyme PSY1 [23].

BBX proteins also play vital roles in response to abiotic stress and in the regulation of phytohormonal signaling in plants. *BBX2* expression is consistently upregulated during long periods of cold stress in *Arabidopsis* [26]. *BBX18* and *BBX23* actively regulate the thermal morphogenesis of *Arabidopsis* by interacting with ELF3 and COP1, while their mutations cause thermo responsive hypocotyl shortening [27]. Heterologous expression of *MdBBX10* in *Arabidopsis* significantly increased the drought and salt tolerance of plants by ABA signaling [28]. Recent studies have shown that the jasmonic acid (JA)-mediated cold stress response can be positively regulated by the BBX37 protein in apple, which is mainly attributed to the synergistic regulation of the BBX37-ICE 1-CBF module by JAZ [29]. In addition, *BBX* family genes function in phytohormone signaling pathways. For example, *AtBBX18* (*AtDBB1a*) accelerates hypocotyl elongation by accumulating the content of gibberellin (GA) [30], whereas *AtBBX20* (*AtBZS1*) negatively regulates the brassinosteroid signaling pathway [31]. MdBBX22 directly interacts with ABI5, the key regulator of the abscisic acid (ABA) signalling pathway, thereby inhibiting MdABI5 transcriptional activity [32]. However, the role of alfalfa *MsBBX* genes in the abiotic stress response and phytohormone signaling pathways remains to be investigated.

Alfalfa (*Medicago sativa* L.) represents the most important and widely distributed legume plant globally, and has been praised for its high protein content, nutritional quality, good palatability and strong adaptability [33]. However, alfalfa growth/development and production are severely restricted by environmental factors, especially water deficiency and salinity stress, thus affecting quality and yield. Although BBX family members play essential roles in many plant species, little research has been conducted on alfalfa *BBX* genes and their roles in various stress responses to date. The publication of alfalfa whole genome data facilitates a comprehensive detailed analysis of genes in the *BBX* family [34]. In the present study, we systematically analyzed the alfalfa *BBX* gene family at the whole genome level, including protein basic information, phylogenetic relationships, chromosomal distributions, gene structures, conserved domains and motifs, and *cis*-regulatory elements. Furthermore, we analyzed transcriptomic data and performed qRT-PCR analysis to investigate the expression of the alfalfa *BBX* genes. The function of the *MsBBX11* gene in salt stress tolerance was identified by heterologous expression in *Arabidopsis*. Our results provide a basis for further exploration of the function of *MsBBX* genes and resistance breeding in alfalfa.

## Results

### Genome-wide identification of ***MsBBX*** genes in alfalfa

To identify the candidate members of *BBX* gene family in alfalfa, 32 AtBBXs and 30 OsBBXs were used as query sequences to screen protein database of alfalfa. After removing redundant sequences and performing domain identification, a total of 28 putative *MsBBX* genes containing the BBX domain (PF00643) were confirmed in the alfalfa genome and were named *MsBBX1* to *MsBBX28* according to their chromosomal position (Table 1). Analysis of the protein physicochemical properties showed that the *MsBBX* family members had an average of approximately 297 amino acids (aa) in length, ranging from 190 (MsBBX21/23) to 436 aa (MsBBX2). The molecular weights (MWs) of the MsBBX proteins ranged from 20.95 (MsBBX21) to 48.73 (MsBBX2) kDa, and the theoretical isoelectric points (pIs) ranged from 4.84 (MsBBX9) to 9.74 (MsBBX4). In addition, the aliphatic indices of the MsBBX proteins ranged from 52.52 (MsBBX6) to 73.80 (MsBBX22). The GRAVY values of all the proteins were negative, ranging from (-0.926) (MsBBX6) to (-0.342) (MsBBX18), implying that the MsBBXs are hydrophilic proteins. Subcellular localization prediction showed that all MsBBXs were located in the nuclei (Table 1).

**Table 1:** Physicochemical properties of 28 *MsBBX* genes identified in the alfalfa genome

Gene name	Gene ID	Chr locus	Peptide residue (aa)	MW (KDa)	pI	Aliphatic index	GRAVY	Subcellular localization
*MsBBX1*	MS.gene33091.t1	Chr1.1	319	35.78	6.27	56.93	-0.691	Nucleus
*MsBBX2*	MS.gene76302.t1	Chr1.1	436	48.73	5.44	61.08	-0.748	Nucleus
*MsBBX3*	MS.gene24218.t1	Chr1.2	275	30.34	6.78	68.44	-0.549	Nucleus
*MsBBX4*	MS.gene065133.t1	Chr1.2	250	27.68	9.74	55.44	-0.662	Nucleus
*MsBBX5*	MS.gene058459.t1	Chr1.4	317	35.56	6.34	58.20	-0.668	Nucleus
*MsBBX6*	MS.gene029402.t1	Chr1.4	353	40.11	6.77	52.52	-0.926	Nucleus
*MsBBX7*	MS.gene94484.t1	Chr2.1	294	32.42	5.03	53.44	-0.618	Nucleus
*MsBBX8*	MS.gene72452.t1	Chr2.3	294	32.36	5.18	53.44	-0.615	Nucleus
*MsBBX9*	MS.gene032179.t1	Chr2.3	243	27.42	4.84	65.51	-0.594	Nucleus
*MsBBX10*	MS.gene014928.t1	Chr3.1	290	31.86	7.11	67.31	-0.482	Nucleus
*MsBBX11*	MS.gene68784.t1	Chr3.2	276	30.62	6.62	67.17	-0.441	Nucleus
*MsBBX12*	MS.gene69436.t1	Chr3.3	237	26.68	6.22	71.98	-0.494	Nucleus
*MsBBX13*	MS.gene014247.t1	Chr3.3	247	27.02	6.36	71.13	-0.406	Nucleus
*MsBBX14*	MS.gene064725.t1	Chr3.4	236	26.70	5.98	72.71	-0.492	Nucleus
*MsBBX15*	MS.gene063133.t1	Chr3.4	277	30.74	6.58	67.29	-0.434	Nucleus
*MsBBX16*	MS.gene003668.t1	Chr4.1	259	29.20	7.09	59.50	-0.612	Nucleus
*MsBBX17*	MS.gene062898.t1	Chr4.2	259	29.06	7.87	61.00	-0.564	Nucleus
*MsBBX18*	MS.gene003876.t1	Chr4.2	240	26.51	4.92	73.58	-0.342	Nucleus
*MsBBX19*	MS.gene015721.t1	Chr4.3	416	47.08	5.14	64.71	-0.685	Nucleus
*MsBBX20*	MS.gene04795.t1	Chr4.4	402	45.57	5.47	64.30	-0.694	Nucleus
*MsBBX21*	MS.gene010593.t1	Chr5.1	190	20.95	8.01	64.21	-0.632	Nucleus
*MsBBX22*	MS.gene68034.t1	Chr5.3	205	22.56	8.01	73.80	-0.485	Nucleus
*MsBBX23*	MS.gene59440.t1	Chr5.4	190	20.96	8.01	62.68	-0.653	Nucleus
*MsBBX24*	MS.gene022048.t1	Chr7.1	401	44.02	5.05	63.07	-0.543	Nucleus
*MsBBX25*	MS.gene23011.t1	Chr7.2	372	41.62	6.13	63.68	-0.705	Nucleus
*MsBBX26*	MS.gene035678.t1	Chr8.1	320	35.42	6.93	61.03	-0.455	Nucleus
*MsBBX27*	MS.gene57909.t1	Chr8.1	343	37.82	6.02	60.90	-0.426	Nucleus
*MsBBX28*	MS.gene012430.t1	Chr8.3	376	41.38	6.02	60.98	-0.410	Nucleus

### Phylogenetic analysis of MsBBX proteins

**Fig. 1:**
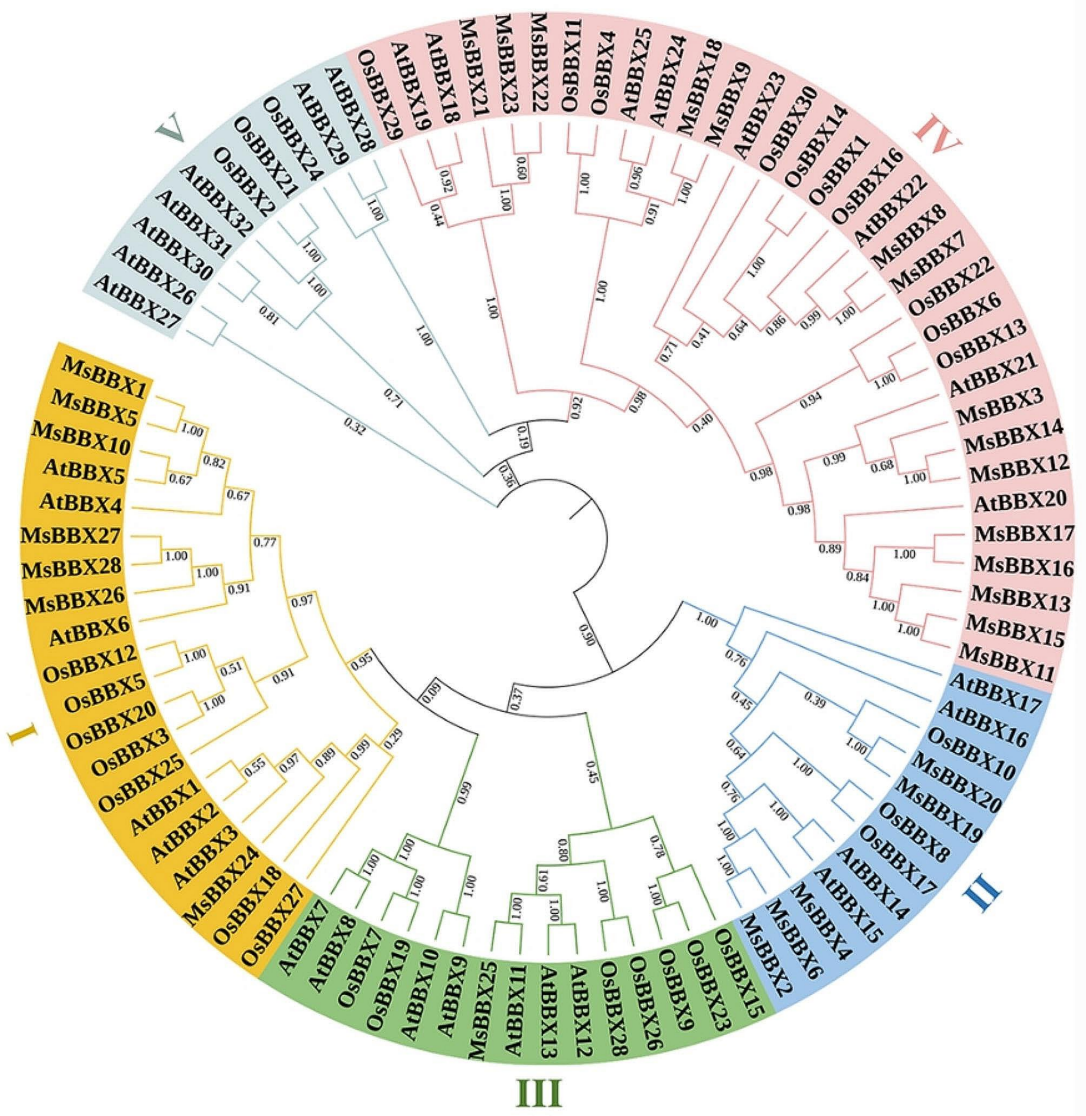
Phylogenetic tree analysis of BBX proteins from alfalfa (MsBBX), *Arabidopsis* (AtBBX) and rice (OsBBX). The tree was divided into five clades represented by different colors. The bootstrap values are indicated at each node

A neighbor-joining tree was constructed using MEGA to investigate the genetic evolution relation of the *MsBBX* gene family based on BBX proteins from alfalfa (28), *Arabidopsis* (32) and rice (30). As shown in Fig. 1, the 90 BBXs were divided into five subfamilies (I-V) depending on the sequence homology, and they were unequally distributed among the five subfamilies. Interestingly, no MsBBX members of alfalfa were found in subfamily V, which had the fewest BBX members with only 10 proteins. The results showed that the largest cluster was subfamily IV with 33 BBX members, including 15 MsBBXs, 8 AtBBXs and 10 OsBBXs. There were 20, 12 and 15 BBX members in subfamilies I, II and III, with 7, 5, and 1 MsBBX members, respectively. Concurrently, BBX proteins from alfalfa, *Arabidopsis* and rice in subfamilies I-IV were grouped into the same clade, suggesting that the BBX family was highly evolutionary conserved and might perform similar biological functions. The phylogenetic tree showed that the BBX proteins of alfalfa were more closely related to their orthologous in *Arabidopsis* than those in rice (Fig. 1).

### Gene structure, motif, and conserved domain analysis of the ***MsBBX*** genes

**Fig. 2:**
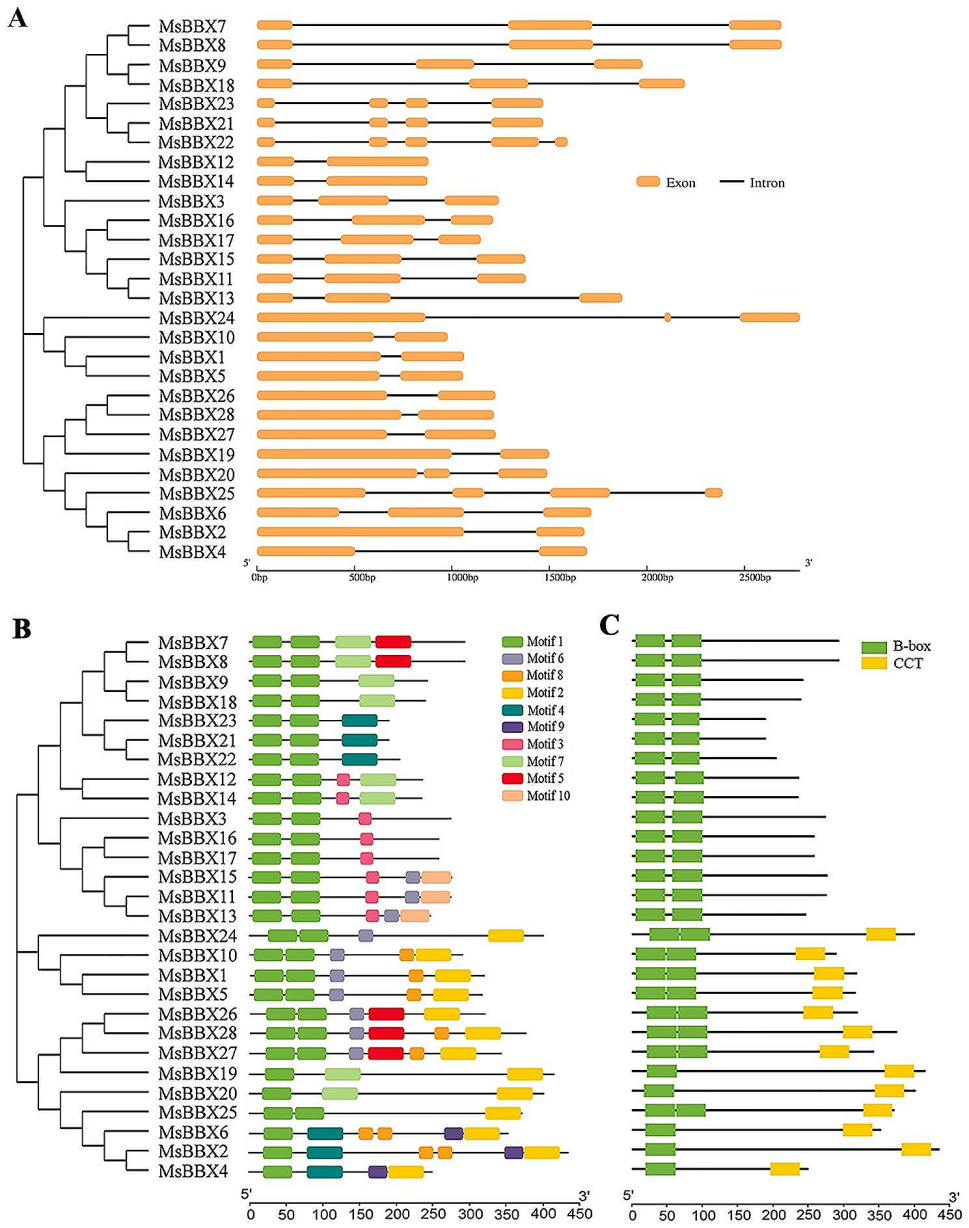
Gene structure, conserved motif and conserved domain analysis of *MsBBX* genes. **A** Exon-intron structure analysis of *MsBBX* genes. The orange boxes and black lines indicate exons and introns, respectively. **B** Motif positions of *MsBBX* genes. Each motif is represented in a colored box. **C** Conserved domain analysis of MsBBX proteins. The green and yellow boxes indicate the B-box and CCT domains, respectively

To explore the structural features of the alfalfa *MsBBX* gene family, the exon-intron structure and conserved motifs were identified. The results showed that there were 2 to 5 exons and 1 to 4 introns in the *MsBBX* genes (Fig. 2A). We found that 11 *MsBBX* genes (39.3%) contained two exons, 13 genes (46.4%) had three exons, and 3 genes (10.7%) had four exons. In particular, *MsBBX22* had five exons and four introns. However, all 28 *MsBBX* genes lacked the UTR region (Fig. 2A). Conserved motif prediction showed that ten distinct motifs (motifs 1–10) were discovered in MsBBX proteins (Fig. 2B). Among them, motifs 1, 2 and 6 were the top three motifs, and were present in 100%, 46.4% and 35.7%, respectively, of the MsBBX proteins, indicating that these motifs are the most critical components of MsBBXs. According to Fig. 2C, motif 1 and motif 2 are the B-box and CCT domains, respectively. The B-box domain was distributed among all the MsBBX proteins, with a total of 23 MsBBXs having two B-boxes, while the other proteins (MsBBX2, MsBBX4, MsBBX6, MsBBX19, and MsBBX20) had only one. In addition, we identified 13 MsBBXs containing a CCT domain (Fig. 2C). The number of motifs in the MsBBXs ranged from 3 to 6. Most of the MsBBX members (11) contained three motifs, and four members had six motifs. Furthermore, the MsBBX proteins that were closely related to the proteins in the phylogenetic tree had similar motif compositions. For instance, MsBBX11, MsBBX13, and MsBBX15 all contained 5 motifs, including motifs 1, 3, 6, and 10. The motif 9 only appeared in MsBBX2, MsBBX4, and MsBBX6 (Fig. 2B). Similar to the composition of motifs, the conserved domains were also distributed according to genetic relationships (Fig. 2C). Multiple sequence alignment showed the conserved domain locations in the MsBBX protein sequences, where all the MsBBXs shared the conserved B-box domain at the N-terminus, and some of the sequences also had a CCT domain at the C-terminus (Fig. S1).

### Chromosome localization, gene duplication and collinearity analysis of ***MsBBX*** genes

**Fig. 3:**
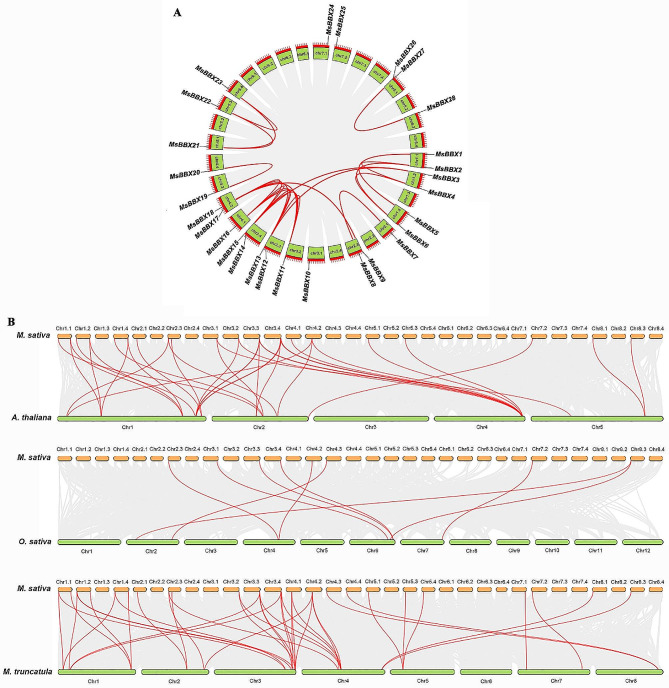
Chromosome distributions of *MsBBX* genes and synteny analysis between *M. sativa* and three other plant species. **A** Chromosomal location and duplication event analysis in the *M. sativa* genome. The segmental duplicated genes are connected by red curves. **B** Synteny analysis between *M. sativa* and *A. thaliana*, *O. sativa*, and *M. truncatula*. The grey lines indicate synteny blocks in *M. sativa* and the other species, while the red lines highlight the collinearity of *BBX* gene pairs

The chromosomal positions and collinearity of the alfalfa *MsBBX* genes were mapped against published genome data. The 28 *MsBBX* genes were unevenly scattered across 20 out of the 32 chromosomes in alfalfa (Fig. S2, Fig. 3A). Each of the eight chromosomes (Chr1.1, 1.2, 1.4, 2.3, 3.3, 3.4, 4.2, 8.1) contained two *MsBBX* genes, and the remaining twelve chromosomes (Chr2.1, 3.1, 3.2, 4.1, 4.3, 4.4, 5.1, 5.3, 5.4, 7.1, 7.2) contained only one *MsBBX* gene. Gene duplication event analysis showed that no tandem duplications occurred in the alfalfa *MsBBX* gene family. Notably, a total of 24 gene pairs exhibited segmental duplication events, and these genes were distributed on chromosomes 1, 2, 3, 4, 5, and 8 (Table S1, Fig. 3A). Most *MsBBX* genes had 1–3 paralogous genes in alfalfa, while five *MsBBXs* (*MsBBX11*, *MsBBX13*, *MsBBX15*, *MsBBX16*, and *MsBBX17*) were found with 4 paralogous genes.

To explore the potential evolutionary relationships of the *BBX* genes between alfalfa and *A. thaliana*, *O. sativa*, or *M. truncatula*, a comparison of collinear maps were constructed. As shown in Fig. 3B and Table S2, a total of 23, 8 and 26 *MsBBX* genes showed syntenic relationships with *Arabidopsis*, *O. sativa*, and *M. truncatula*, respectively. Among these *MsBBXs*, 36, 9, and 40 pairs of orthologous genes were found between alfalfa and *Arabidopsis*, *O. sativa*, and *M. truncatula*, respectively. Most *MsBBX* genes were identified with 1–2 orthologous gene pairs in *Arabidopsis*, while *MsBBX12* and *MsBBX14* had three orthologous gene pairs. Except for *MsBBX10* and *MsBBX27*, all the other *MsBBX* genes displayed syntenic relationships with *M. truncatula*, and *MsBBX3* and *MsBBX16* had three orthologous genes. Moreover, the largest number of collinear gene pairs was observed between alfalfa and *M. truncatula*, suggesting that the BBX proteins were highly conserved between the two legumes (Fig. 3B).

### Analysis of ***cis***-regulatory elements in ***MsBBX*** gene promoters

**Fig. 4:**
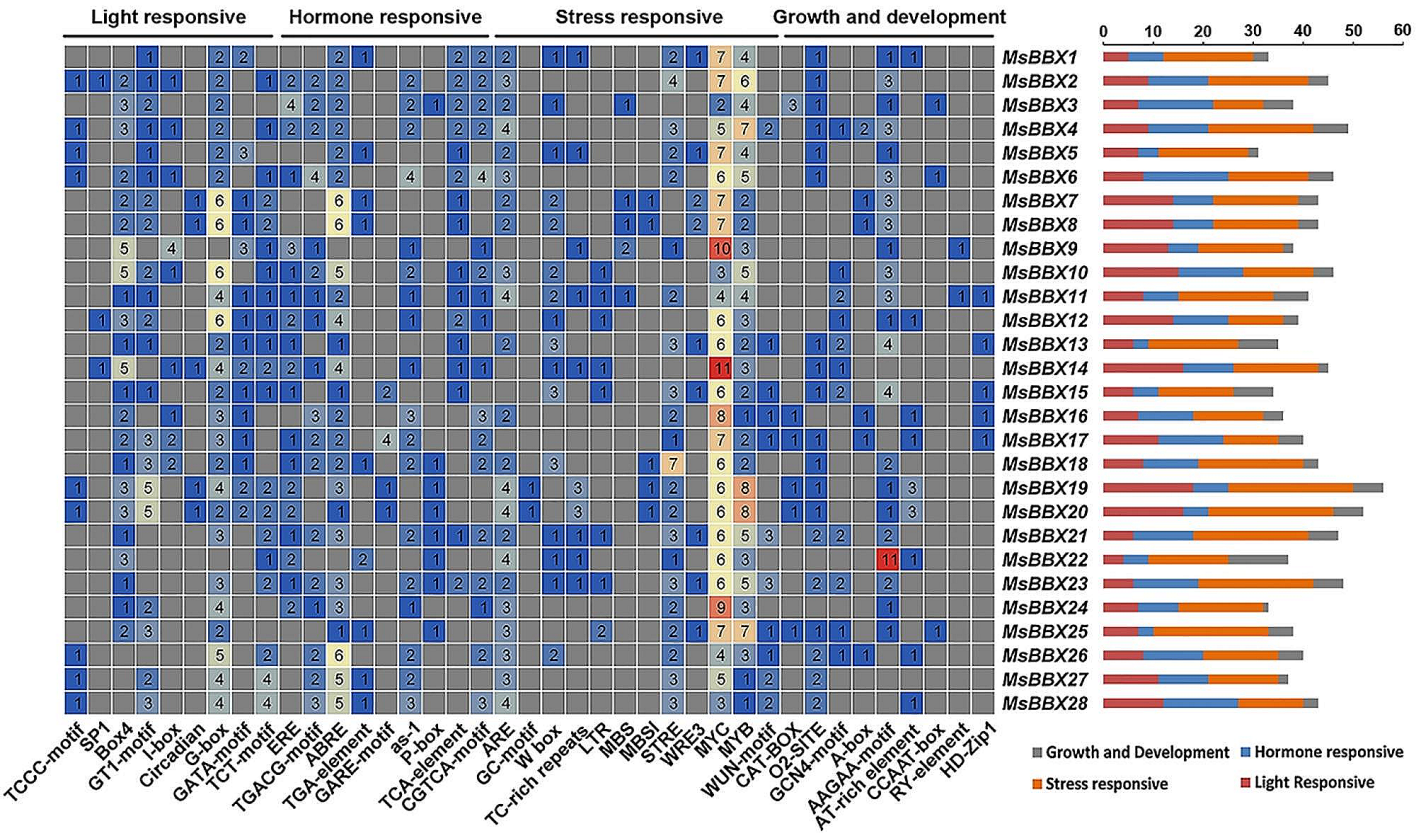
Cis-regulatory element analysis of *MsBBX* genes. The gradient colors in the grid represent the number of *cis*-regulatory elements in the *MsBBXs*. The multicolor histogram indicates the number of different categories of *cis*-elements in these *MsBBX* genes

To better explore the potential regulatory mechanism of the *MsBBX* gene family, the *cis*-regulatory elements in the promoter sequences (2000 bp upstream of the start codon) of the *MsBBX* genes were analyzed using PlantCARE. Results showed that 39 types of *cis*-regulatory elements in the *MsBBX* promoter regions, with 12 (31%) related to stress response, nine (23%) related to growth and development, nine (23%) related to light responsiveness, and nine (23%) related to phytohormone response, respectively (Fig. 4, Table S3). All the *MsBBXs* contained these four categories of *cis*-regulatory elements. Stress-responsive elements were the most abundant elements, with MYC and MYB elements present in all the *MsBBX* family genes, ranging from 2 to 11 and 1 to 8, respectively. In addition, more than 78% of the *MsBBX* genes contained ARE and STRE of stress-related *cis*-elements (Fig. 4). In particular, stress and light response elements were most common in the *MsBBX19* gene. The phytohormone-related elements identified in the *MsBBX* genes were associated with MeJA-responsive, ABA-responsive, IAA-responsive, GA-responsive, and SA-responsive (Table S3). Importantly, the ABRE involved in the ABA response, TGACG-motif (CGTCA-motif) involved in the JA response, and ERE involved in the ET response, appeared 77, 35 and 32 times in 26, 18 and 19 *MsBBX* genes, respectively, accounting for more than 64% of phytohormone responsive genes. Moreover, 87 and 54 G-box and Box4 elements involved in light responsiveness were found in 26 and 23 *MsBBX* genes, respectively. These results suggest that *MsBBXs* may play a key role in the alfalfa response to different environmental stresses and plant growth regulators.

### Expression profiles of ***MsBBX*** genes in different tissues in alfalfa

In order to study the expression patterns of *MsBBX* genes in alfalfa, we analyzed the transcriptome data of leaves, flowers, pre-elongated stems, elongated stems, roots and nodules in the NCBI database (Fig. S3). In the database, 15 *MsBBX* genes were found in different tissues of alfalfa, while no relevant information was found for the remaining 13 *MsBBX* genes. As shown in Fig. S3, most of the *MsBBXs* were highly expressed in leaves, flowers, pre-elongated stems and elongated stems, while their expression levels were lowest in nodules, suggesting that they play a role in the development of aboveground tissues. Interestingly, the expression level of *MsBBX14* was the highest in the root tissues, indicating that *MsBBX14* plays crucial roles in root development. Five *MsBBX* genes (*MsBBX7*, *MsBBX15*, *MsBBX18*, *MsBBX21*, and *MsBBX27*) were highly expressed in the flowers. *MsBBX9* was more highly expressed in leaves than in other plant tissues, whereas the maximum expression of *MsBBX10* and *MsBBX17* occurred in pre-elongated stems. These results suggest that the *MsBBX* genes have tissue-specific expression profiles and functions during alfalfa development.

### Expression profiles of ***MsBBX*** genes in alfalfa under different abiotic stresses

To further explore the expression profiles of *MsBBX* members under abiotic stress conditions, we downloaded the transcriptome data for alfalfa plants treated with drought and salt from the NCBI, and performed RNA-seq analysis. As shown in Fig. S4A, most of the *MsBBX* genes were positively induced by drought stress. The transcription levels of 8 *MsBBXs* (*MsBBX7*, *MsBBX8*, *MsBBX11*, *MsBBX12*, *MsBBX16*, *MsBBX20*, *MsBBX26*, and *MsBBX28*) peaked at 1 h and then decreased gradually with increasing drought duration. *MsBBX2*/*18* and *MsBBX4*/*21* were significantly upregulated at 3 and 12 h, respectively. However, *MsBBX14* and *MsBBX19* expression levels were significantly downregulated after drought stress. In addition, *MsBBX15* expression was unaltered during drought treatment (Fig. S4A). Under salt treatment, the expression of most of the *MsBBX* transcripts changed except for that of *MsBBX15* and *MsBBX21* (Fig. S4B). The transcript levels of 21 *MsBBX* genes were upregulated to different degrees under salt treatment at different times. For instance, the transcript levels of *MsBBX12*, *MsBBX16*, *MsBBX17*, *MsBBX20* and *MsBBX26* significantly increased after 0.5 h of salt treatment, while the transcript levels of *MsBBX2*, *MsBBX4*, *MsBBX11*, *MsBBX27* and *MsBBX28* markedly increased after 1 h. The peak expression levels of *MsBBX3* were observed after 24 h of salt stress. The expression of four *MsBBX* genes (*MsBBX6*, *MsWBBX13*, *MsBBX14* and *MsBBX19*) decreased differentially during salt treatment (Fig. S4B). These results indicate that *MsBBX* genes may be involved in drought and salt stress responses in alfalfa.

**Fig. 5:**
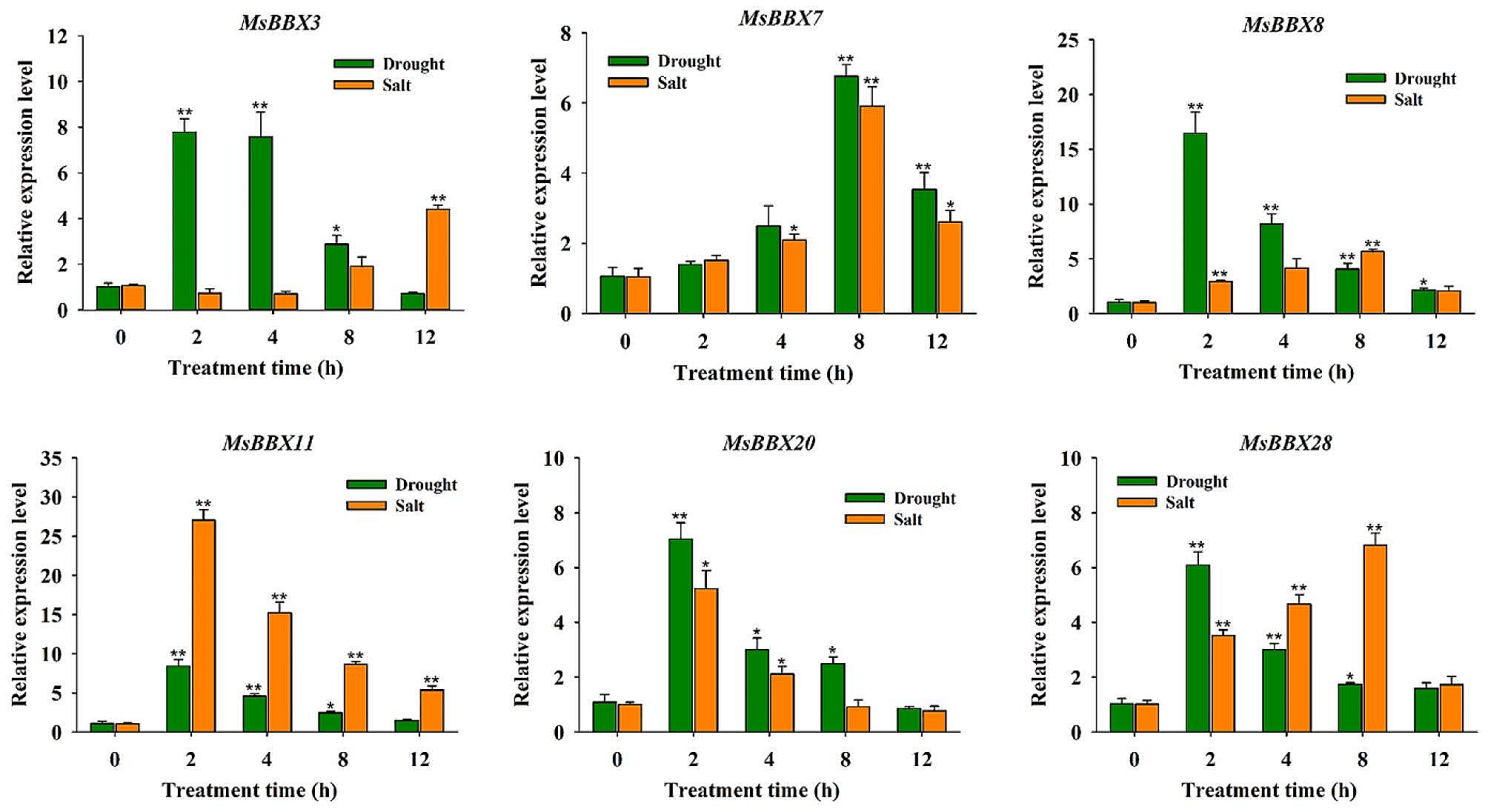
Expression analysis of six selected genes under drought and salt stresses by qRT-PCR. Values are the means ± SEs (*n* = 9). Asterisks and double asterisks above the bars indicate significant differences: *P < 0.05; **P < 0.01

We randomly selected six *MsBBX* genes (*MsBBX3*, *MsBBX7*, *MsBBX8*, *MsBBX11*, *MsBBX20*, and *MsBBX28*) that responded positively to drought and salt stress for qRT-PCR verification. As shown in Fig. 5, the six *MsBBX* genes exhibited diverse expression patterns during drought and salt stress treatments, and the patterns were largely consistent with the results of the transcriptome analysis. All the selected *MsBBX* genes were strongly induced by drought stress, and their expression was strongly elevated and peaked at 2 or 8 h. These *MsBBX* genes were also strongly induced by salt stress, and the expression levels reached a maximum at 2, 8 or 12 h (Fig. 5). In particular, the *MsBBX11* gene was upregulated 27-fold at 2 h of salt stress treatment compared to the 0 h. Except for the *MsBBX3* gene, the expression levels of all the selected *MsBBX* genes initially increased and subsequently decreased under salt stress. These results indicate that these *MsBBX* genes may participate in drought and salt adaptation in alfalfa.

### Analysis of ***MsBBX*** gene expression in response to plant growth regulator treatments in alfalfa

To identify hormone-responsive *MsBBXs*, we investigated the expression of *MsBBX* family genes in alfalfa treated with ABA at different times using RNA-seq data. The expression of most *MsBBX* genes was induced at different levels under ABA treatment (Fig. S5). Twenty-two *MsBBX* genes exhibited a positive response to ABA treatment, of which the expression of 17 *MsBBXs* first increased and then decreased. In particular, the expression of *MsBBX11/12/17/22/28* and *MsBBX4*/*15*/*24* increased dramatically after 1 and 3 h of ABA treatment, respectively. Compared with the control treatment, ABA treatment caused a gradual decrease in the transcript levels of *MsBBX9*, *MsBBX13*, *MsBBX14* and *MsBBX18* compared to 0 h. The expression level of *MsBBX21* remained unchanged during ABA treatment (Fig. S5).

**Fig. 6:**
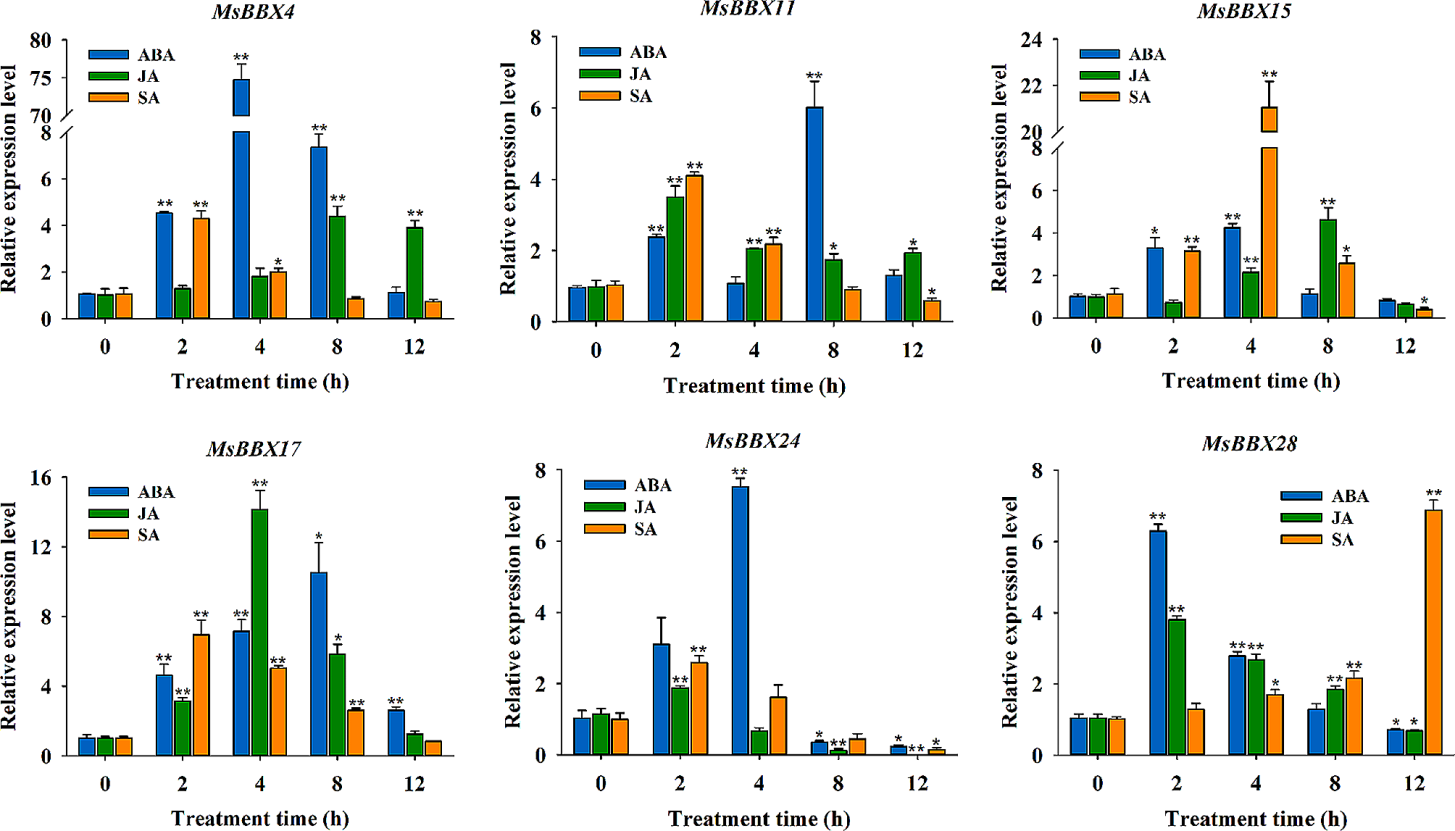
Expression analysis of six selected genes under ABA, JA and SA treatments by qRT-PCR. Values are the means ± SEs (*n* = 9). Asterisks and double asterisks above the bars indicate significant differences: *P < 0.05; **P < 0.01

According to the RNA-seq analysis, six genes (*MsBBX4*, *MsBBX11*, *MsBBX15*, *MsBBX17*, *MsBBX24* and *MsBBX28*) that positively responded to ABA treatment were analyzed by qRT-PCR at 0 h, 2 h, 4 h, 8 h, and 12 h after ABA, JA and SA treatments to investigate the response of *MsBBX* genes to plant growth regulators (Fig. 6). The expression of all the selected *MsBBX* genes was significantly induced by ABA, JA and SA at different treatment time intervals. The transcript levels of *MsBBX4*, *MsBBX15* and *MsBBX24* peaked after 4 h of ABA treatment, and that of *MsBBBX4* increased almost 75-fold compared with that at 0 h. *MsBBX11*, *MsBBX17* and *MsBBX28* were highly induced in response to ABA treatment at 2 or 8 h. The expression trends of these *MsBBX* genes under ABA treatment were consistent with the transcriptome analysis results. All six *MsBBX* genes responded positively to JA treatment and reached maximum expression at 2, 4 or 8 h (Fig. 6). The expression level of the *MsBBX* genes showed a trend of first increasing and then decreasing under JA treatment conditions. *MsBBX17* was highly expressed at 4 h and upregulated by 14-fold compared with that at 0 h. With the exception of *MsBBX28*, the expression of the selected genes initially increased and subsequently decreased under SA treatment. The expression of the *MsBBX28* gene increased significantly (approximately 7-fold) at 12 h compared with that in the control. Notably, *MsBBX15* was strongly induced in response to SA treatment at 4 h, reaching approximately 21-fold greater expression than that at 0 h (Fig. 6).

### Protein-protein interactions among the MsBBXs

To explore the comprehensive functions of MsBBXs in alfalfa, a protein interaction network was generated based on homologous proteins from *Arabidopsis* using the STRING database. A total of 12 MsBBX proteins were predicted to interact with each other (Fig. S6). The results showed that MsBBX23 had the most interactions with MsBBX proteins (seven), followed by MsBBX18 which interacted with six MsBBX proteins. MsBBX5 and MsBBX22 both have five interacting proteins and interact with each other. In addition, MsBBX2, MsBBX25 and MsBBX27 interacted separately with one MsBBX protein each, namely MsBBX22, MsBBX1 and MsBBX23, respectively. These results indicate that MsBBXs may function through interactions.

### Subcellular localization of the MsBBX proteins

**Fig. 7:**
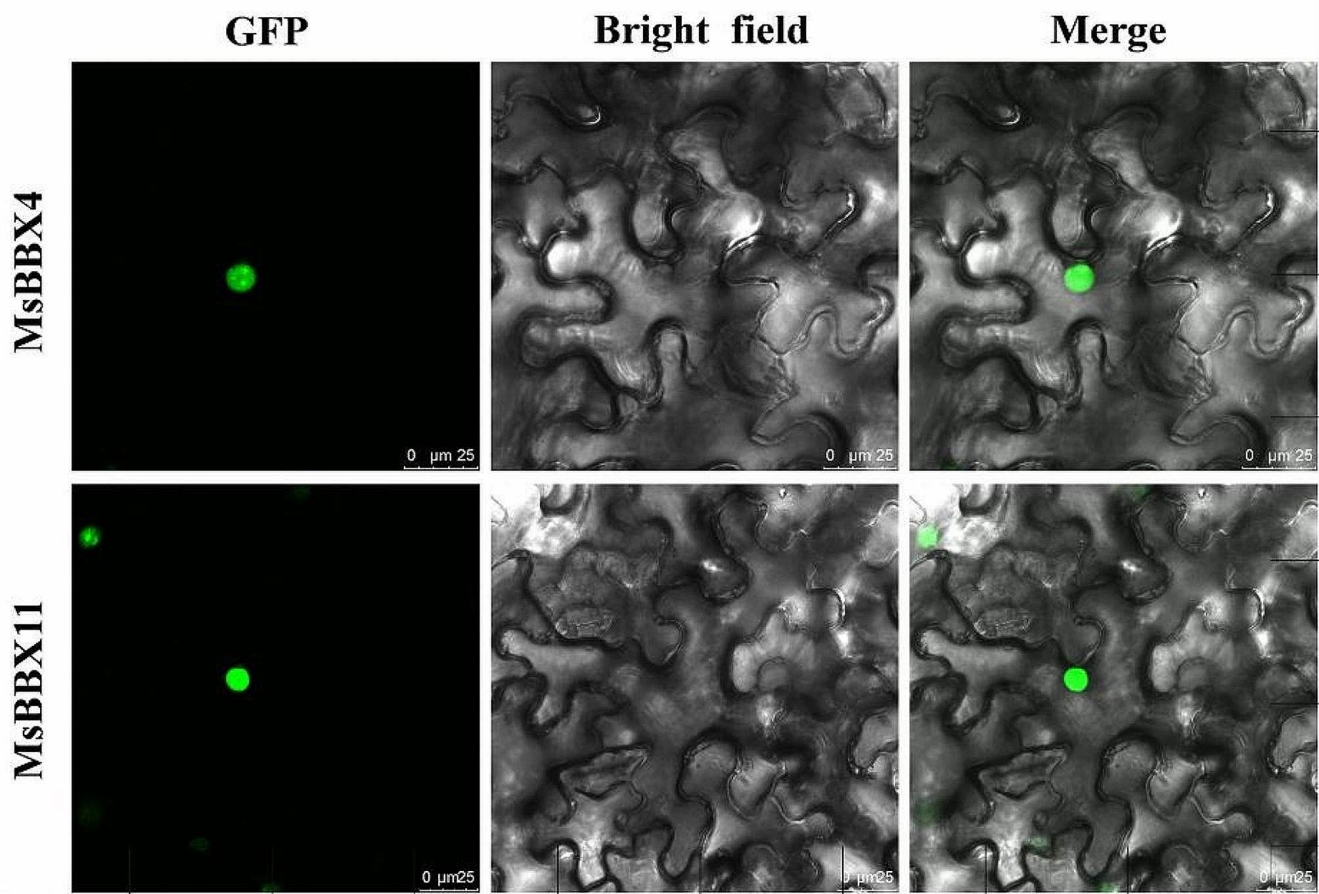
Subcellular localization of the MsBBX4 and MsBBX11 proteins. Images from left to right represent green fluorescent protein (GFP), bright field and an overlay (GFP and bright field) from the same sample. Scale bar = 25 μm

Prediction of the subcellular localization of MsBBX proteins using Plantm-PLoc revealed that all the MsBBXs were localized in the nuclei (Table 1). To further verify the prediction results and understand the functions of MsBBXs, we selected two *MsBBX* genes (*MsBBX4* and *MsBBX11*) that were strongly induced by abiotic stress or plant growth regulators for transient expression in tobacco leaves. The results showed that the green fluorescent signals expressed by the MsBBX4-GFP and MsBBX11-GFP fusion vectors could be observed only in the nuclei, which was consistent with the predicted results (Fig. 7). These results suggest that MsBBX4 and MsBBX11 encode nuclear-localized proteins.

### Overexpression of ***MsBBX11*** in ***Arabidopsis*** confers tolerance to salt stress

**Fig. 8:**
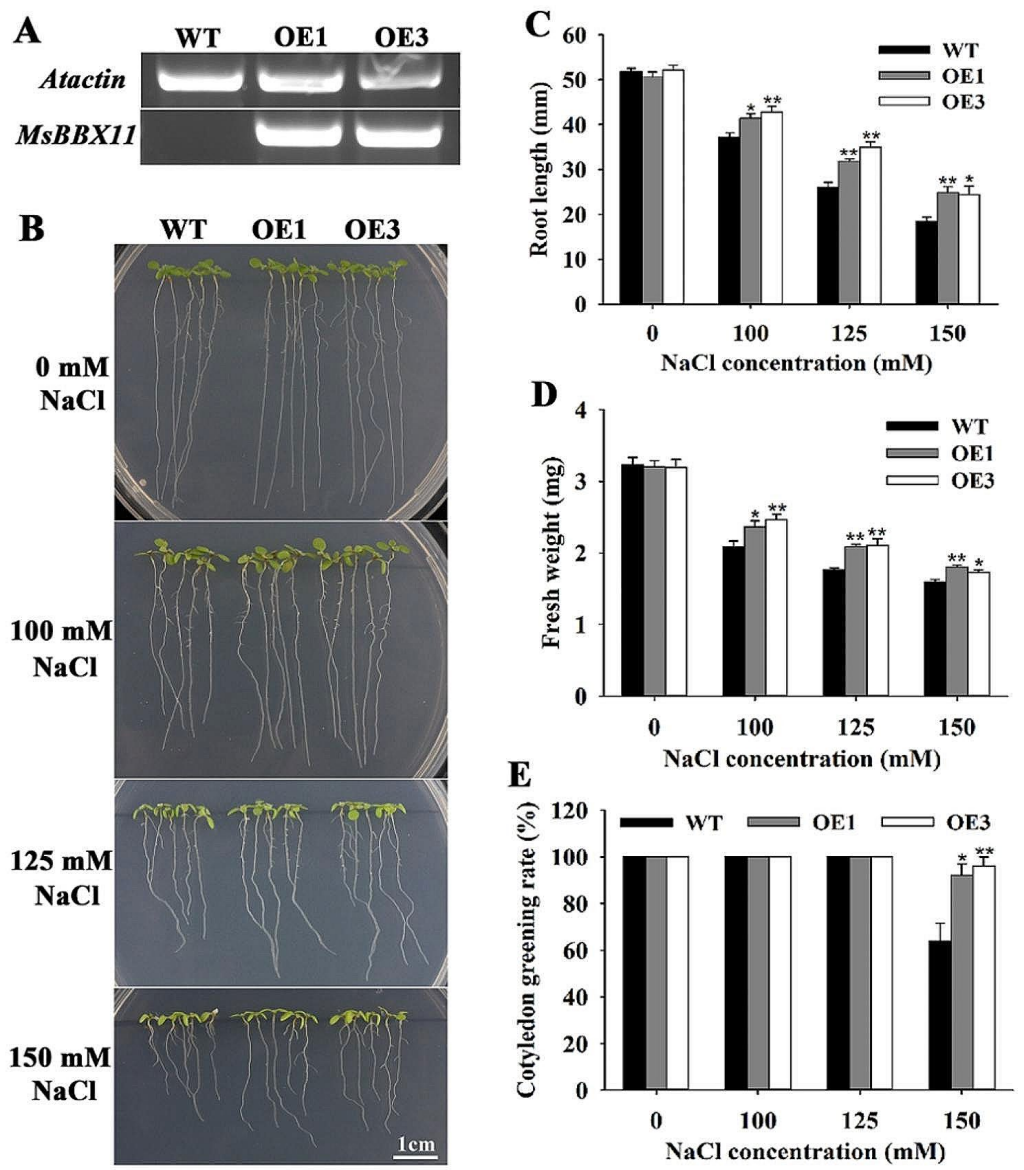
Overexpression of *MsBBX11* enhanced the salt tolerance of *Arabidopsis* during the seedling stage. **A** Semi-quantitative RT-PCR analysis of *MsBBX11* expression levels in WT and transgenic lines. **B** Root growth phenotypes of *Arabidopsis* WT and transgenic seedlings vertically grown on MS medium supplemented with 0, 100, 125 or 150 mM NaCl. **C** Analysis of root length. **D** Analysis of fresh weight. **E** Analysis of cotyledon greening rate. Values are the means ± SE of three biological replicates. Asterisks and double asterisks above the bars indicate significant differences: *P < 0.05; **P < 0.01

To reveal the biological roles of the *MsBBX* genes, we selected a gene with high expression under salt stress, *MsBBX11*, from the qRT-PCR data of alfalfa for further study. *MsBBX11* transgenic *Arabidopsis* plants were obtained by PPT screening and confirmed by semi-quantitative RT-PCR analysis. Two homozygous lines (OE1 and OE3) were randomly selected for further salt tolerance assays. Semi-quantitative RT-PCR result showed that *MsBBX11* expression was detected in OE1 and OE3 lines but not in WT plants (Fig. 8A, Fig. S7). As shown in Fig. 8B, salt stress inhibited the root growth of both WT and transgenic lines. However, after 100, 125 or 150 mM NaCl stress, the transgenic plants exhibited higher primary root length than the WT (Fig. 8C). Correspondingly, the fresh weight of the transgenic plants was significantly higher than that of WT plants (Fig. 8D). Moreover, the OE1 and OE3 lines showed significantly higher cotyledon greening rates than the WT under 150 mM NaCl treatment (Fig. 8E).

**Fig. 9:**
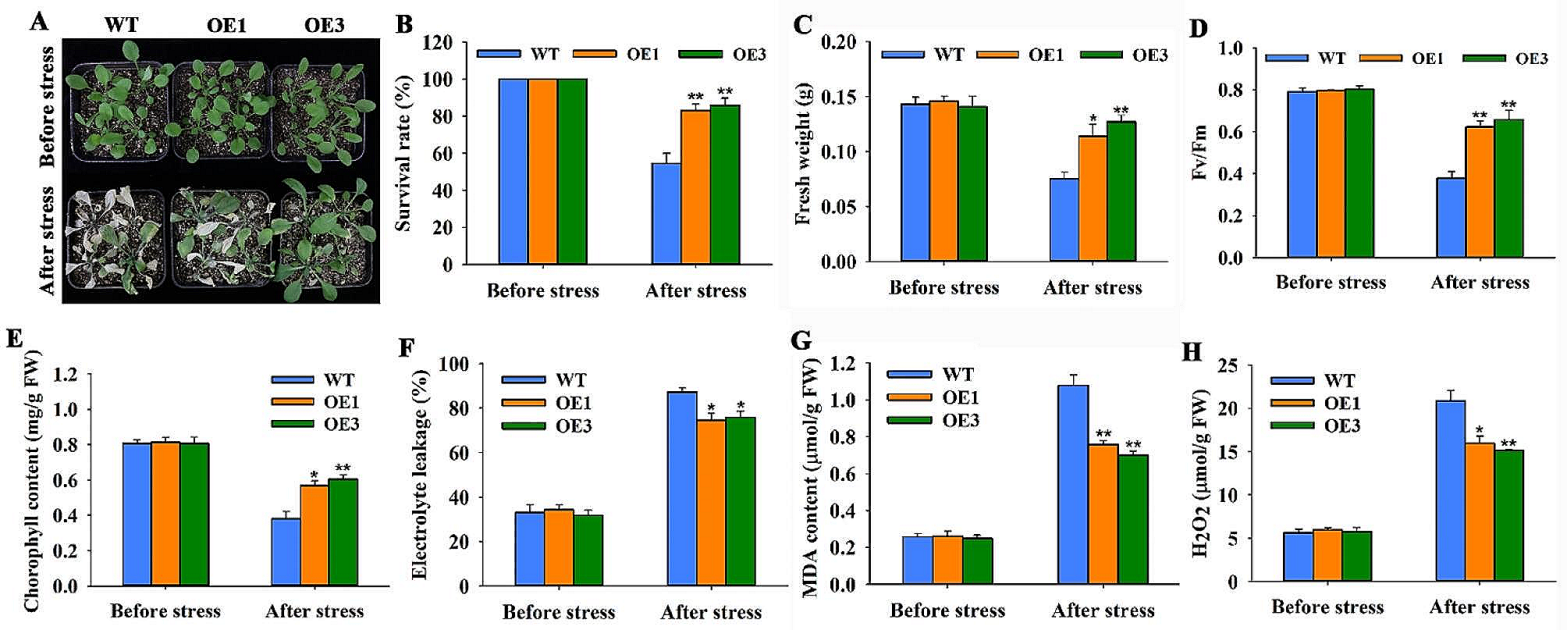
Overexpression of *MsBBX11* enhanced the salt tolerance of *Arabidopsis* at the vegetative stage. **A** Growth performance of the WT and transgenic plants before and after 300 mM NaCl treatment. Survival rate **B**, fresh weight **C**, *Fv/Fm***D**, chlorophyll content **E**, electrolyte leakage **F**, MDA content **G**, and H_2_O_2_ accumulation **H** of the WT and transgenic plants before and after salt treatment. Values are the means ± SEs of three biological replicates. Asterisks and double asterisks above the bars indicate significant differences: *P < 0.05; **P < 0.01

To elucidate the role of *MsBBX11* in salt resistance in soil, *MsBBX11*-overexpresing plants and WT were exposed to 300 mM NaCl stress for 10 days. As shown in Fig. 9A, there was no obvious difference in morphology between the transgenic plants and WT under normal growth conditions. However, upon exposure to NaCl, the WT plants exhibited more conspicuous leaf damage than the transgenic lines (Fig. 9A). The survival rates of OE1 and OE3 lines were 83.2% and 85.8%, respectively, while only 54.5% of the WT plants survived (Fig. 9B). Moreover, the fresh weight, *Fv/Fm* ratio, and chlorophyll content of the transgenic plants were higher than those of the WT plants under salt stress (Fig. 9C-E). Salt stress increased electrolyte leakage and the accumulation of MDA and H_2_O_2_, but these effects were significantly greater in WT than in transgenic plants (Fig. 9F-H). There were no significant differences in these indicators between the WT and transgenic lines under normal growth conditions.

## Discussion

BBX proteins belong to a super family of zinc-finger TFs that participate in plant growth, development, and response to abiotic stress and phytohormones [7]. To date, whole genome identification of *BBX* family members has been performed extensively in dicots and monocots, for example, *Arabidopsis* [4], *O. sativa* [8], tomato [9], tobacco [35] and *Malus domestica* [10]. However, the identification and functional analysis of the *BBX* gene family in alfalfa have not been reported. In this study, we used the whole genome sequence to perform a systematic bioinformatic identification and functional analysis of the *BBX* gene family in alfalfa. The results of this study will provide valuable information for further investigations of the functions of *MsBBX* members in alfalfa and will provide candidate genes for alfalfa stress tolerance breeding.

In this study, we identified 28 *MsBBX* family members from the alfalfa genome. Ma et al. [36] counted the number of *BBX* genes in 13 different plant species and approximately 30 *BBX* family members in each, indicating that the number of *BBX* genes in these plants, including alfalfa, was relatively stable. In contrast, there are 64 *BBX* genes in apple [10] and 19 in millet [11]. Yin et al. [37] reported that there was no direct relationship between the number of *BBX* family genes and the plant genome size, and we speculate that the remarkable variation may be caused by species-specific duplications or deletions during evolution. Previous studies have shown that the *BBX* genes in various plants are typically classified into 5 subfamilies [4, 11, 37]. In the present study, BBX proteins were divided into 5 subfamilies (I-V) according to sequence similarity to *Arabidopsis*, rice and alfalfa BBX proteins (Fig. 1). However, no alfalfa MsBBX proteins were grouped into subfamily V, indicating that the evolution of alfalfa MsBBX family may be different from that of other plants. A similar phenomenon was found in tobacco plants [35].

The diversity of gene structures typically plays an important role in the evolution of multiple gene families. *BBX* family genes contain one or two conserved B-box domains, and some possess a CCT domain in plants [4]. We also found similar results for *MsBBX* family genes (Fig. 2), indicating that the BBXs are relatively conserved among different species. Based on gene structure and motif analysis, the *MsBBX* genes in the same groups had similar intron/exon combinations and motif compositions (Fig. 2), suggesting that they may have similar biological functions. Generally, gene duplication events are the main drivers of new gene emergence and genome evolution. Tandem and segmental replication are the two main duplication patterns in plants [38]. In alfalfa, a total of 24 pairs of segmental duplications were found in the *MsBBX* gene family, but no tandem duplications occurred (Fig. 3A), implying that segmental duplications were particularly beneficial for the expansion of *MsBBX* family members. Similar results were reported by Ma et al. [3] during the evolution of the *PeBBX* gene family. In addition, many isogenous gene pairs were detected between alfalfa and *Arabidopsis*, *O. sativa*, and *M. truncatula* (Fig. 3B), suggesting the indispensable role of these genes in the evolution of the *BBX* family.

BBX proteins are functionally diverse in regulating plant growth, development and stress responses, which is further supported by the detection of numerous hormone- and stress-related elements in the promoter regions of the tomato and tobacco *BBX* genes [9, 35]. Promoter *cis*-regulatory elements regulate the transcription of specific genes in response to stress signals by binding to transcription factors [39]. In the present study, promoter elements associated with growth and development, the stress response and plant growth regulator response were found to be abundant among the *MsBBX* genes (Fig. 4), indicating that the *MsBBX* genes may actively participate in these physiological processes and stress resistance. In *Arabidopsis*, BBX32 can regulate the flowering pathway via interaction with CONSTANS-LIKE 3 (COL3)/BBX4 [40]. The direct interaction between BBX32 and BBX21 suppresses BBX21-HY5 and thus functions in light signaling [41]. A protein interaction network analysis suggested that the MsBBX proteins might synergistically regulate the biological processes of alfalfa through interactions.

Previous studies have reported that *BBX6*/*COL5* accelerated *Arabidopsis* flowering by activating the transcription of FT under short-day conditions [18], while in contrast, *BBX32*/*EIP6* regulated flowering in a manner independent of CO under long day conditions [42]. According to the transcriptome data of alfalfa, most *MsBBXs* were related to the growth and development of aboveground tissues in alfalfa (Fig. S3). Among them, the expression levels of *MsBBX7*/*15*/*18*/*21*/*27* varied greatly among the flowers, indicating that these genes might play a critical role in the regulation of flower development. In addition, Ma et al. [36] found that *CaBBX5* and *CaBBX6* are involved in photomorphogenesis and are highly expressed highly in leaves. Similarly, *MsBBX9* and *MsBBX17* exhibited relatively high transcriptional activity in alfalfa leaves and stems, respectively, while *MsBBX14* was highly expressed in roots, suggesting their potential involvement in seedling morphogenesis.

Although *BBX* genes have diverse functions, we concerned about their response to abiotic stress. Previous studies have reported that nine *VvBBX* genes were significantly upregulated in response to drought stress in berry [43]. In alfalfa, we found that most of the *MsBBX* genes positively responded to drought or salt stress (Fig. S4A, S4B), suggesting that these genes may have potential functions in plant drought or salt tolerance. The *MdBBX10* gene has been proven to enhance the drought and salt tolerance of transgenic *Arabidopsis* [28]. This study revealed that several *MsBBXs* were responsive to both drought and salt stress, as verified by the qRT-PCR results for the six selected genes (Fig. 5). Therefore, it can be assumed that the *MsBBX* genes are positive regulators of drought and salt stress signaling in alfalfa.

Previous studies have shown that the transcription of *AtBBX24* is positively related to salt stress signaling, and that the overexpression of *AtBBX24* significantly increases salt stress resistance in *Arabidopsis* [44]. In this study, we further validated the function of the *MsBBX11* gene, which actively responded to salt stress. It was found that overexpression of *MsBBX11* in *Arabidopsis* promoted seedling growth and photosynthetic capacity, and reduced cell membrane damage and H_2_O_2_ accumulation (Figs. 8 and 9), thus conferring salt tolerance to the plants. The difference in transgene expression is an important factor determining the effectiveness of transgenic transformation, and is usually influenced by the sequence flanking the insertion site or other factors [45]. The expression level of the OE1 plants was relatively lower than that of the OE3 plants, and this difference might be related to the positional effect of the transgene or the specific insertion mode [45].

Plant *BBX* genes are also involved in hormone signal transduction. In *Arabidopsis*, BBX21 physically interacts with the HY5 or ABI5 proteins to repress ABI5 expression, thereby negatively regulating the inhibition of seed germination by ABA [46]. Recent studies have revealed that the BBX22-ABI5 interaction module negatively regulates chlorophyll degradation and leaf senescence through an ABA-dependent pathway [32]. MdBBX37 positively regulates JA-mediated cold stress tolerance through the JAZ-BBX37-ICE1-C BF pathway in apple [29]. In pepper, five *BBX* genes were significantly induced by SA treatment [36]. Most *MsBBXs* were upregulated under ABA treatment in alfalfa (Fig. S5). In addition, the expression of six selected genes (*MsBBX4*, *MsBBX11*, *MsBBX15*, *MsBBX17*, *MsBBX24* and *MsBBX28*) was dramatically induced by ABA, JA and SA treatments (Fig. 6), which corresponded to phytohormone response elements in their promoter regions. It has been previously reported that *MdBBX10* enhances abiotic stress tolerance through ABA signaling [28]. In this study, the *MsBBX11* and *MsBBX28* genes were positively induced by drought, salt, and plant growth regulator treatments simultaneously. However, whether *MsBBXs* regulate abiotic stress through hormone signaling remains to be further explored. Therefore, we speculate that *MsBBXs* may serve as positive regulators of ABA, JA, and SA signal transduction, participating in regulation of growth and abiotic stresses in alfalfa.

## Conclusions

In the present study, 28 *MsBBX* genes were systematically explored in alfalfa and phylogenetically grouped into four subfamilies. The discovery of duplication and collinearity gene pairs provided valuable information about the evolutionary history of the *MsBBX* genes. We discovered that the *MsBBX* genes exhibit tissue specificity and that most *MsBBXs* may play important roles in aboveground tissue development. Furthermore, the expression of several *MsBBX* genes was significantly induced by drought, salt and hormone stress, suggesting that *MsBBXs* play essential roles in plant stress response. For instance, the *MsBBX11* gene markedly improved the salt tolerance of transgenic *Arabidopsis* and can be used as a candidate gene for salt tolerance breeding in alfalfa. It will be of great interest to investigate the biological functions of these *MsBBX* genes and elucidate their detailed regulatory mechanisms in the future. This study lays an important foundation for creating stress resistant germplasms and breeding new varieties of alfalfa in the future.

## Materials and methods

### Plant materials

The alfalfa cultivar ‘Zhongmu No. 1’ was used in this study, and its seeds were provided by the Institute of Animal Science, Chinese Academy of Agricultural Sciences, China. Seeds of tobacco (*Nicotiana benthamiana*) and *Arabidopsis thaliana* Columbia-0 wide type (WT) used in this study were preserved in our laboratory (Laboratory of Forage Molecular Breeding, Ningxia University, China).

### Genome-wide identification of alfalfa ***BBX*** genes

We downloaded the whole genome and annotation files of alfalfa from the website https://figshare.com/projects/whole_genome_sequencing_and_assembly_of_Medicago_sativa/66380 [34]. The reported BBX protein sequences of *Arabidopsis thaliana* and *Oryza sativa* were acquired from the TAIR website (https://www.arabidopsis.org/) and Phytozome13 database (https://phytozome-next.jgi.doe.gov/), respectively [47]. These proteins were queried against *BBX* gene family members from alfalfa in the BLASTP search. The Hidden Markov Model (HMM) profile of the B-box domain (PF00643) was used as the seed sequence to search the alfalfa genome [8]. After manually removing the redundant sequences, the candidate *MsBBX* family genes were further identified using the Conserved Domains Database (CDD) (http://www.ncbi.nlm.nih.gov/cdd/), SMART (https://smart.embl-heidelberg.de/) and Pfam database comparison (http://pfam.xfam.org/) [48].

### Protein property analysis and phylogenetic analysis

TBtools software was used to acquire the genomic positions of the corresponding *MsBBX* genes from the alfalfa genome data [49]. The physical and chemical properties of the MsBBX proteins were predicted by ExPASy website (http://web.expasy.org/protparam/) [50]. Subcellular localization of the MsBBX proteins was determined by Plantm-PLoc (http://www.csbio.sjtu.edu.cn/bioinf/plant-multi/) [51]. Phylogenetic relationships of the BBX proteins among alfalfa, *Arabidopsis* and rice were analyzed based on the neighbor-joining method (1,000 bootstraps) with MEGA 7.0 software [52]. The image of the phylogenetic tree was then beautified using iTOL online software (https://itol.embl.de/) [53].

### Gene structure, conserved motif, domain analysis and multiple sequence alignments

The exon-intron structure of *MsBBX* genes was obtained from the online website GSDS: http://gsds.gao-lab.org/ [54]. Conserved motifs of MsBBXs were identified using Multiple Expectation Maximization for Motif Elicitation (MEME) software [55]. The number of repetitions was set to any, the width of the conserved sites was restricted between 6 and 50, and the maximum number of motifs was set to 10. In addition, the conserved domains of the MsBBX proteins were analyzed by CDD databases (http://www.ncbi.nlm.nih.gov/cdd/). The ClustalW program was used to perform multiple sequence alignments of these MsBBX proteins [56].

### Chromosomal localization and gene duplication analysis of the ***MsBBX*** gene family

The chromosomal localization information of *BBX* genes was retrieved from the genome files of different plants and visualized using TBtools software. The *MsBBX* gene duplication events and collinearity of *BBX* genes in alfalfa, *Arabidopsis*, rice and *Medicago truncatula* were determined by MCScanX software.

### Cis-regulatory element and protein-protein interaction analysis of the ***MsBBX*** gene family

The sequences 2000 bp upstream of the start codon (ATG) of the *MsBBX* genes were extracted from the alfalfa genome database. The PlantCARE online tool (http://bioinformatics.psb.ugent.be/webtools/plantcare/html/) was used to analyze the *cis*-regulatory elements [57]. The *cis*-acting element diagrams of *MsBBX* genes were drawn with TBtools software. The STRING database was used to predict the protein-protein interaction network of the *MsBBX* family genes based on their homologous in *Arabidopsis* [58].

### Expression profiles of ***MsBBX*** genes with transcriptome data

We downloaded the transcriptome data for various tissues and stress treatments in alfalfa from the NCBI database (SRP055547, SRR7091780-7091794, and SRR7160313-7160357) [59–61]. This study analyzed six tissues including leaf, flower, pre-elongated stem, elongated stem, root and nodule and three abiotic stresses, salt, drought and ABA. The differential gene expression analysis was conducted using DESeq2 with |log_2_(fold change)| ≥ 1 and FDR < 0.01. TBtools software was used to complete the heatmap of *MsBBX* gene expression.

### Plant growth conditions and treatments

Plants of the alfalfa cultivar ‘Zhongmu No. 1’ were grown hydroponically in a growth chamber at 23–26 °C and a photoperiod of 16 h light/8 h dark. After four weeks of incubation, the seedlings with consistent growth stages were separated into six groups: (1) control, (2) drought, (3) salt, (4) ABA, (5) JA, and (6) SA. The alfalfa seedlings were subsequently treated with Hoagland solution containing PEG6000 (20%), NaCl (200 mM), ABA (10 μM), SA (100 μM) or JA (100 μM) for 0, 2, 4, 8 or 12 h. Three independent replicates were set for each treatment time point (including control). After treatment, the leaves of alfalfa were frozen in liquid nitrogen and stored at -80 °C for gene expression analysis.

### Gene expression pattern analysis of ***MsBBX*** genes by qRTPCR

The Eastep® Super total RNA Extraction kit (Promega, Shanghai, China) was used to extract the total RNA from each sample. RNA was reverse transcribed and first-strand cDNA was synthesized using a reverse transcription kit (Vazyme, Nanjing, China). qRT-PCR was carried out using ChamQ SYBR qPCR Master Mix (Vazyme, Nanjing, China) and the *MsActin2* gene was used as an internal control [62]. All samples were run in three biological replicates, and each included three technical replicates. Relative expression levels of *MsBBX* genes were calculated using the 2^−∆∆Ct^ method. The primer sequences and melting curves of the *MsBBX* genes are shown in Table S4 and Fig. S8.

### Subcellular localization analysis

For subcellular location assays, the full-length of coding sequences without the stop codon of two selected *MsBBX* genes (*MsBBX4* and *MsBBX11*) were amplified (specific primers are shown in Table S4) and cloned into the pCAMBIA1300-GFP vector, generating the pCAMBIA1300-MsBBX4/11-GFP fusion plasmids. The successfully constructed plasmids were subsequently introduced into *A. tumefaciens* strain GV3101, which was transiently expressed in tobacco leaves [63]. After 2 days of incubation in the dark, the GFP fluorescence signal was captured by a laser confocal microscope (Leica TCS SP8, Germany).

### Plant transformation and transgenic plant generation

We transformed the *A. tumefaciens* strain GV3101 carrying the pCAMBIA1300-MSBX11-GFP recombinant vector into wild-type *Arabidopsis* using the floral dip method to obtain transgenic plants. Transformed *Arabidopsis* with overexpression of *MsBBX11* were selected for 10 mg/L DL-phosphinothricin (PPT) and a total of 17 independent lines were generated. The homozygous transformants (T3) were further confirmed by semi-quantitative RT-PCR using specific primers (Table S4) and two lines (OE1 and OE3) were randomly selected for salt tolerance analysis.

### Salt stress tolerance assays

For salt tolerance analysis of seedlings root elongation, *Arabidopsis* WT and transgenic lines seeds were grown vertically on 1/2 MS agar plates for 7 d and then transplanted to NaCl-containing 1/2 MS plates (0, 100 or 150 mM NaCl) for salt treatment. The primary root length of seedlings was determined after 7 days of growth. For the soil salinity tolerance assay, 7-day-old seedlings were transplanted into soil and watered with Hoagland solution. After two weeks of growth, the plants were irrigated with 300 mM NaCl solution for 10 d for salt treatment [64]. Thereafter, the plant phenotypes were photographed and the survival rates were calculated. The fresh weight of the rosette leaves was measured with a balance. The maximum quantum yield (*Fv/Fm*) of the leaves was measured after 30 min in the dark. The chlorophyll content was determined by 80% acetone according to Liu et al. [65]. Electrolyte leakage was analyzed according to Dahro et al. [66]. The malondialdehyde (MDA) content was measured using thiobarbituric acid (TBA) according to Puckette et al. [67]. The accumulation of hydrogen peroxide (H_2_O_2_) was spectrophotometrically determined according to Jiang and Zhang [68].

### Statistical analysis

The data in the experiment was reported as means ± standard errors (SEs). The statistical significant differences between the control and treatment groups were determined by Student’s *t*-test at 5% (*P < 0.05) or 1% (**P < 0.01) probability levels with SPSS Statistical 20.0 software. Figures were produced using Sigmaplot software (version 12.5).

### Electronic supplementary material

Below is the link to the electronic supplementary material.

**Supplementary Material 1: Table S1.** Paralogous gene pairs in segmental duplication events of alfalfa *MsBBX* genes. **Table S2.** Collinear genes of *MsBBXs* between alfalfa and *Arabidopsis*, alfalfa and *O. sativa*, alfalfa and *M. truncatula*. **Table S3.** The functions of *Cis*-regulatory elements in *MsBBX* gene promoters. **Table S4.** The primers used in this study

**Supplementary Material 2: Fig. S1.** Multiple sequence alignments of the conserved domains of the MsBBX proteins. **Fig. S2.** Distribution and location of the *MsBBX* gene family on alfalfa chromosomes. **Fig. S3.** Transcriptome ananlysis of the expression patterns of the *MsBBX* genes in six tissues of alfalfa: leaf, flower, pre-elongated stem, elongated stem, root and nodule. **Fig. S4.** Expression profiles of the *MsBBX* genes in alfalfa under drought and salt stress from transcriptome data. **Fig. S5.** Expression profiles of the *MsBBX* genes in alfalfa under ABA treatment from transcriptome data. **Fig. S6.** Predicted protein-protein interaction networks of MsBBX proteins based on the interactions of their orthologs in *Arabidopsis*. **Fig. S7.** Semi-quantitative RT-PCR gel image of *MsBBX11* expression levels in WT and transgenic lines (OE1 and OE3). **Fig. S8.** Melting curves for all primers used in the qRT-PCR assays.

## Data Availability

All data generated or analyzed during the present study are available in the submitted manuscript and its supplementary material. The reference genome data and annotation information of alfalfa (Xinjiangdaye) were obtained from figshare data repository (https://figshare.com/projects/whole_genome_sequencing_and_assembly_of_Medicago_sativa/66380). The Arabidopsis and rice BBX protein sequences were downloaded from the TAIR (https://www.arabidopsis.org/) and Phytozome13 database (https://phytozome-next.jgi.doe.gov/), respectively. Transcriptome data for various tissues of alfalfa were downloaded from the NCBI database (SRP055547). Transcriptome data of alfalfa treated with salt, drought and ABA were downloaded from the NCBI database (SRR7091780-7091794 and SRR7160313-7160357).
