# Supplementary material for: Genome-wide identification of B-box zinc finger (BBX) gene family in Medicago sativa and their roles in abiotic stress responses
Source: BMC Genomics. 2024 Jan 24;25:110. doi: 10.1186/s12864-024-10036-4 (PMC10809573; doi:10.1186/s12864-024-10036-4)
Supplement: Supplementary file 2 — Supplementary Material 2: Fig. S1. Multiple sequence alignments of the conserved domains of the MsBBX proteins. Fig. S2. Distribution and location of the MsBBX gene family on alfalfa chromosomes. Fig. S3. Transcriptome ananlysis of the expression patterns of the MsBBX genes in six tissues of alfalfa: leaf, flower, pre-elongated stem, elongated stem, root and nodule. Fig. S4. Expression profiles of the MsBBX genes in alfalfa under drought and salt stress from transcriptome data. Fig. S5. Expression profiles of the MsBBX genes in alfalfa under ABA treatment from transcriptome data. Fig. S6. Predicted protein-protein interaction networks of MsBBX proteins based on the interactions of their orthologs in Arabidopsis. Fig. S7. Semi-quantitative RT-PCR gel image of MsBBX11 expression levels in WT and transgenic lines (OE1 and OE3). Fig. S8. Melting curves for all primers used in the qRT-PCR assays. [file 12864_2024_10036_MOESM2_ESM.docx]

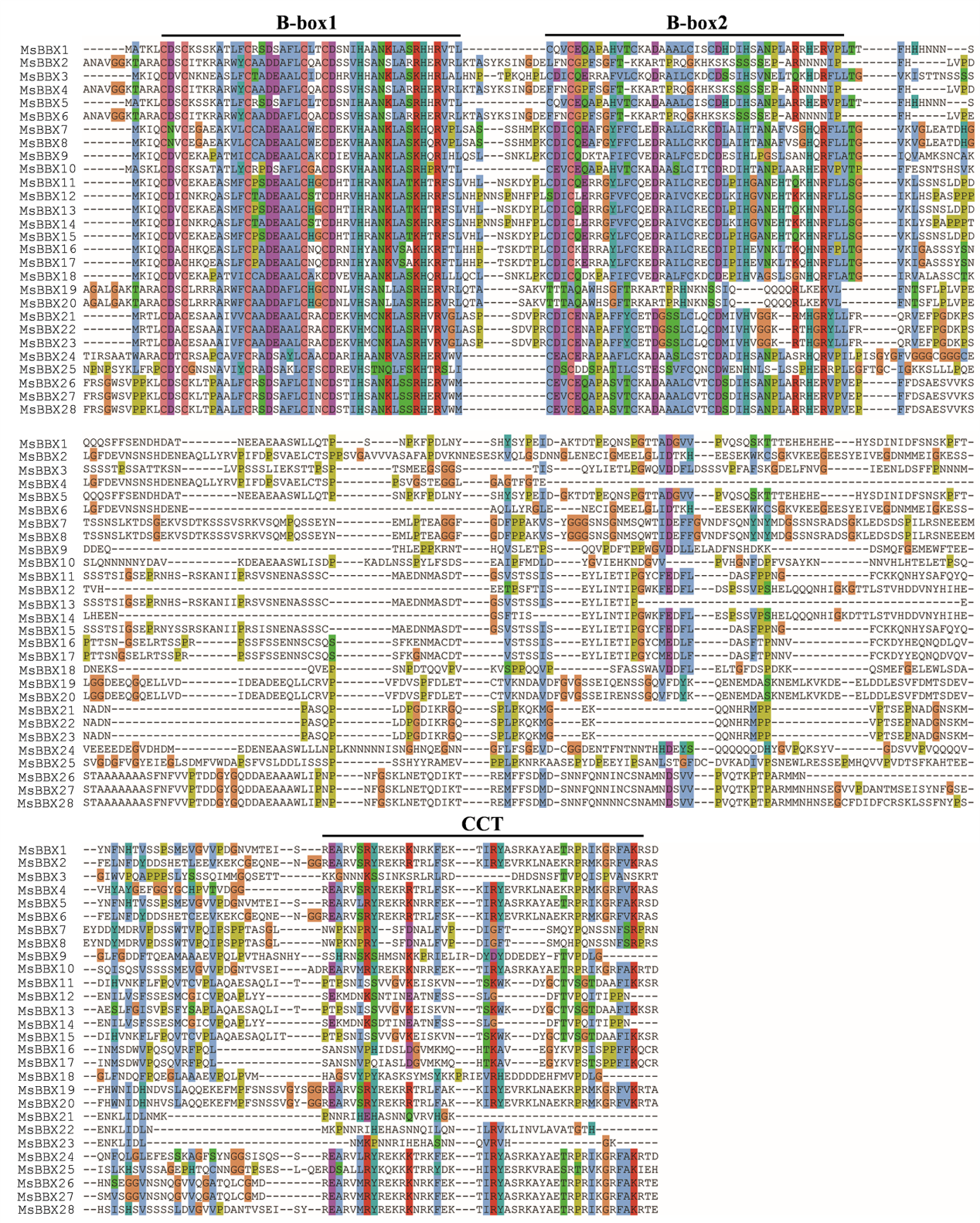


**Figure S1.** Multiple sequence alignments of the conserved domains of the MsBBX proteins. The bold black lines represent the B-box1, B-box2 and CCT domains, and the domain names are shown at the top of the sequence. Protein sequence names are shown on the left. The conserved amino acid residues are shaded in color


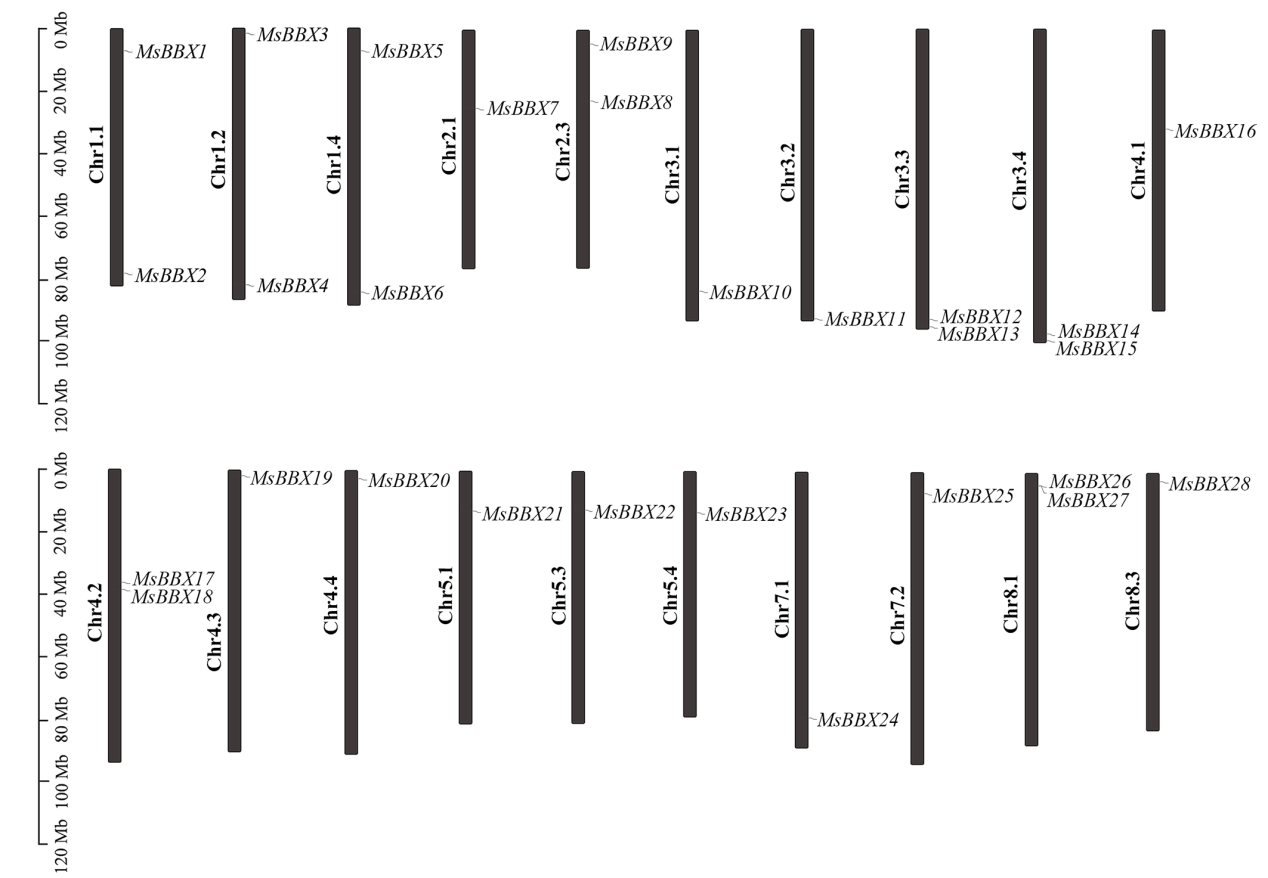


**Figure S2.** Distribution and location of the *MsBBX* gene family on alfalfa chromosomes. The black columns represent the different chromosomes of alfalfa, with the chromosome number labeled on the left side of each column. The black lines indicate the locations of each *MsBBX* gene


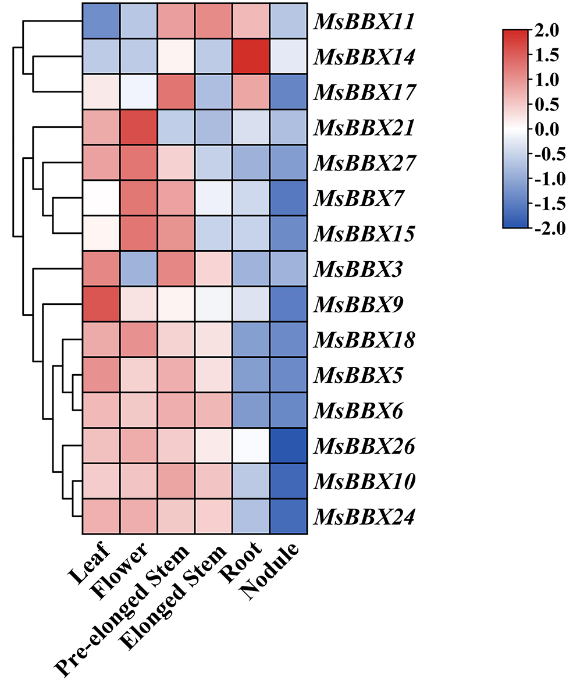


**Figure S3.** Transcriptome ananlysis of the expression patterns of the *MsBBX* genes in six tissues of alfalfa: leaf, flower, pre-elongated stem, elongated stem, root and nodule. The blue and red colors indicate lower and higher transcript abundances, respectively


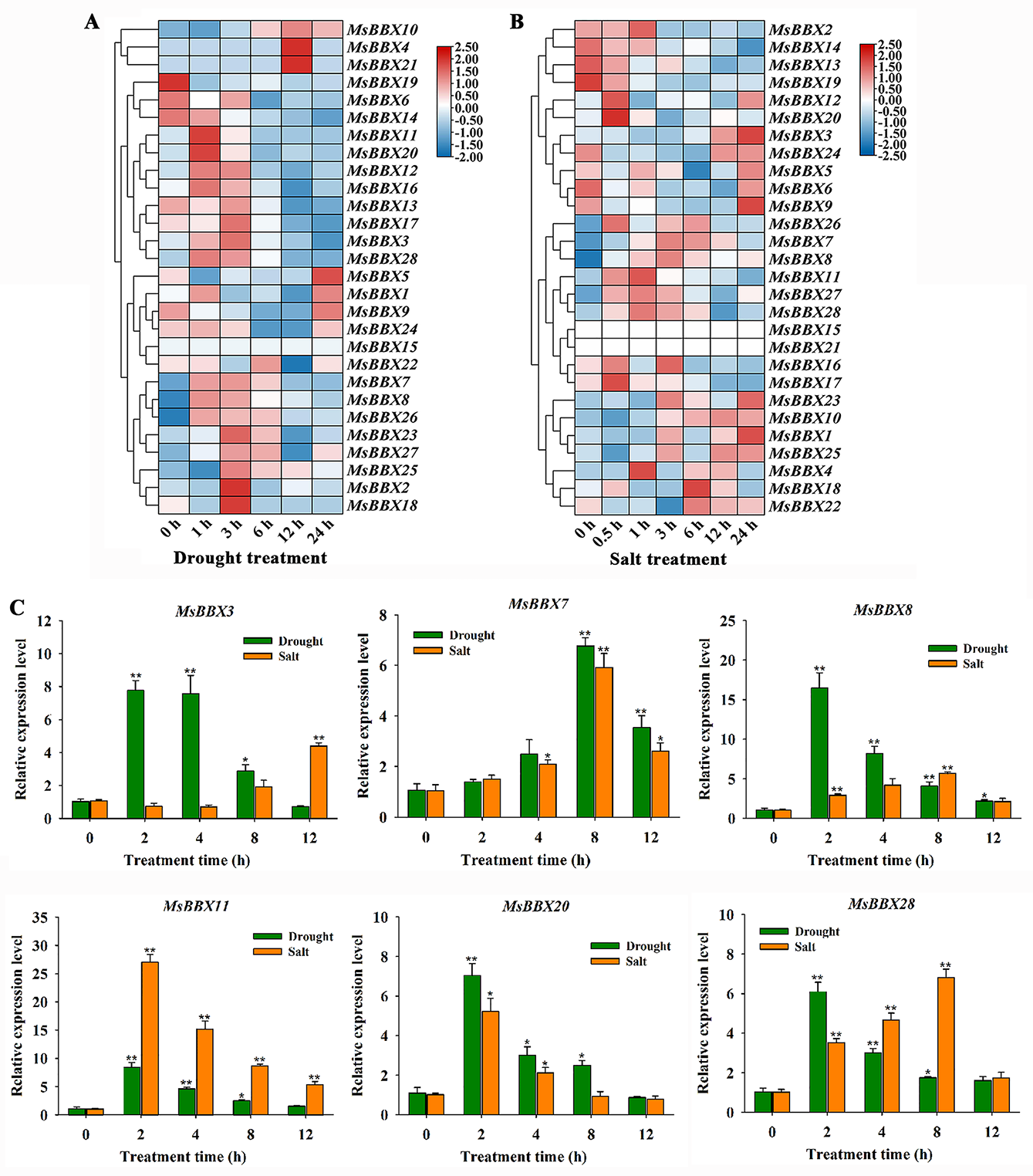


**Figure S4.** Expression profiles of the *MsBBX* genes in alfalfa under drought and salt stress from transcriptome data. **A** RNA-seq analysis of the *MsBBX* genes under drought stress. **B** RNA-seq analysis of the *MsBBX* genes under salt stress. The blue and red colors indicate lower and higher transcript abundances, respectively


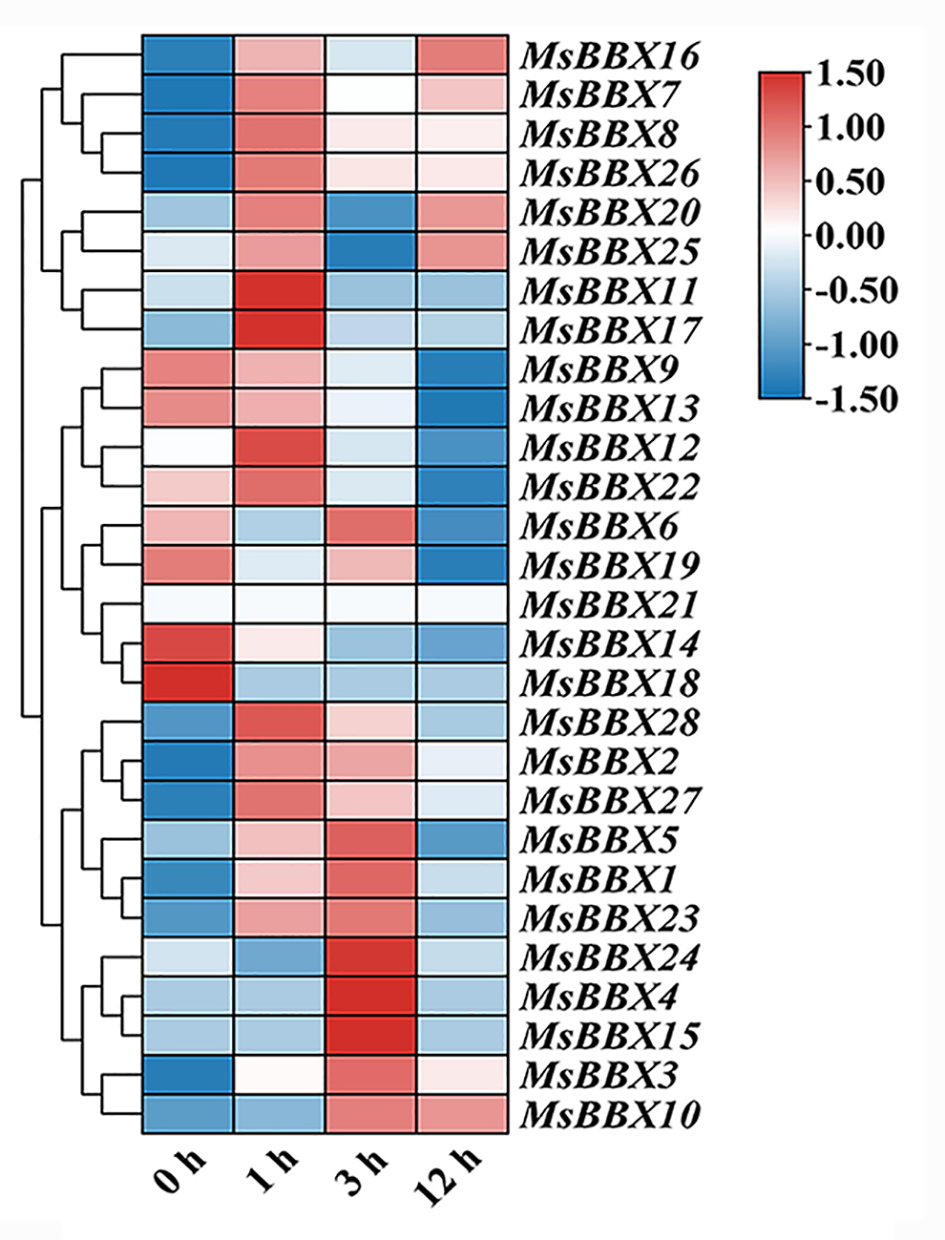


**Figure S5.** Expression profiles of the *MsBBX* genes in alfalfa under ABA treatment from transcriptome data. The blue and red colors indicate lower and higher transcript abundances, respectively


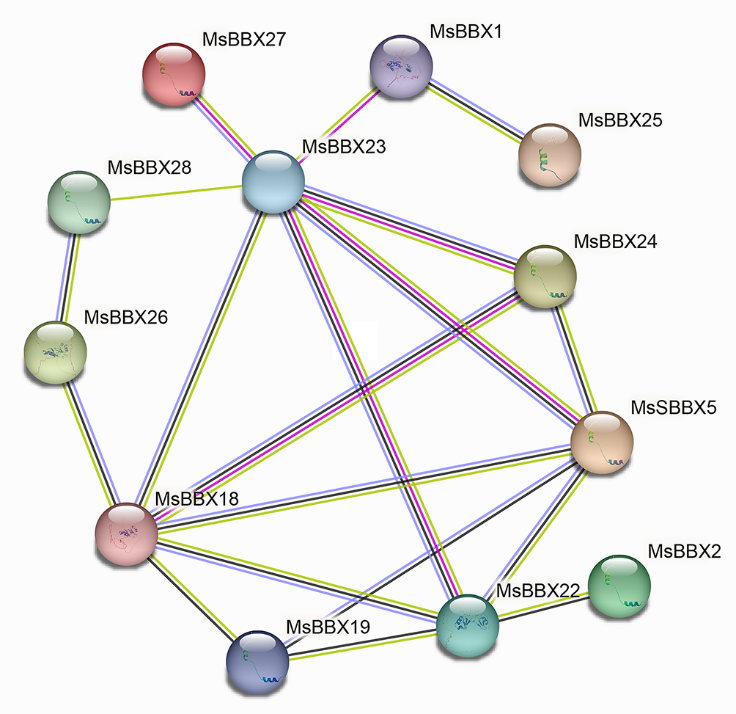


**Figure S6.** Predicted protein-protein interaction networks of MsBBX proteins based on the interactions of their orthologs in *Arabidopsis*. Line and node colors represent different types and degrees of interaction, respectively


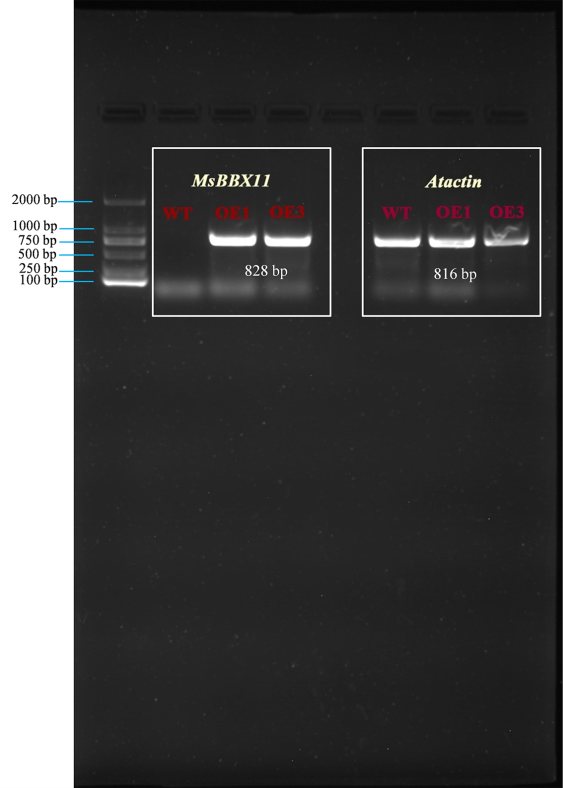


**Figure S7.** Semi-quantitative RT-PCR gel image of *MsBBX11* expression levels in WT and transgenic lines (OE1 and OE3). The gel electrophoresis bands on the left are the 2000 bp marker. The two white boxes represent the gel bands for expression detection of *MsBBX11* and *Atactin* genes in WT and transgenic lines, respectively


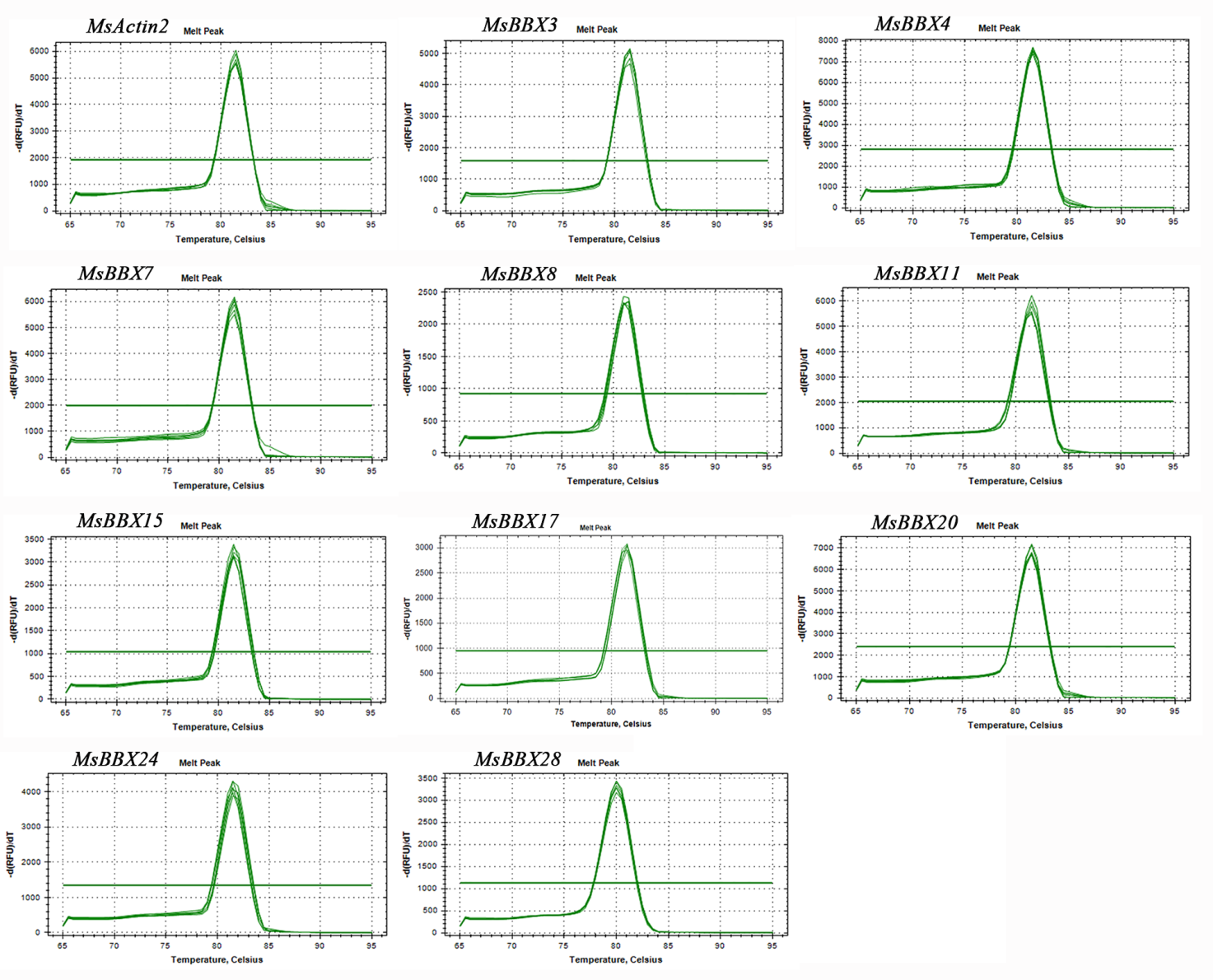


**Figure S8.** Melting curves for all primers used in the qRT-PCR assays. *MsActin2* was used as a reference gene. *MsBBX3*, *MsBBX4*, *MsBBX7*, *MsBBX8*, *MsBBX11*, *MsBBX15*, *MsBBX17*, *MsBBX20*, *MsBBX24*, and *MsBBX28* were genes detected under drought, salt or hormone treatment conditions
